# Supplementary material for: Translocation of vaginal microbiota is involved in impairment and protection of uterine health
Source: Nat Commun. 2021 Jul 7;12:4191. doi: 10.1038/s41467-021-24516-8 (PMC8263591; doi:10.1038/s41467-021-24516-8)
Supplement: Supplementary file 1 — Supplementary Information [file 41467_2021_24516_MOESM1_ESM.pdf]

Supplementary Figures

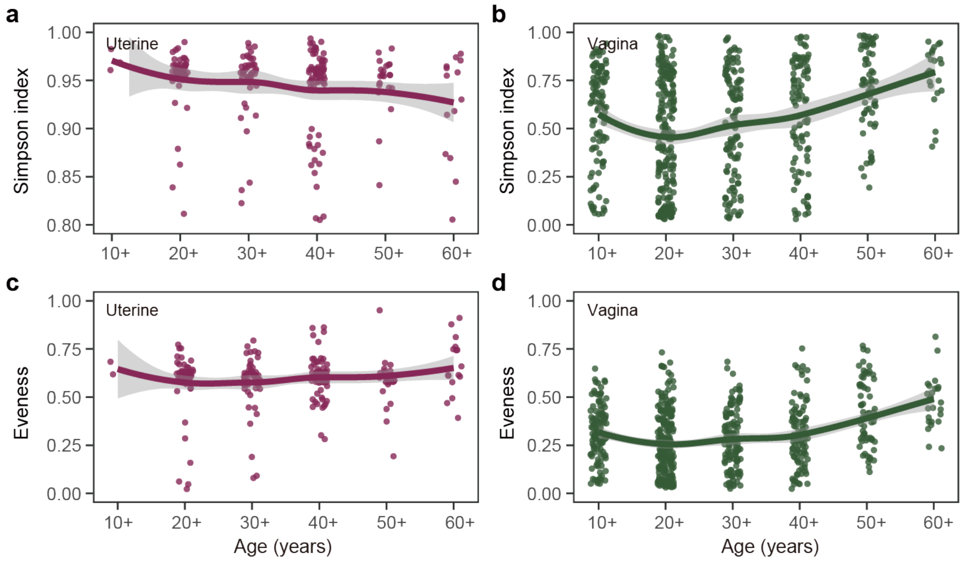

Supplementary Fig. 1 Simpson index (a-b) and evenness (c-d) of the uterine and vaginal microbiota in women of different ages. The scales 10+, 20+, 30+, 40+, 50+, and 60+ on the x-axis represent ages <20, 20-29, 30-39, 40-49, 50-59, and ≥60 years old, respectively. The shadow around the linear regression trendline shows a 95% confidence interval (CI).

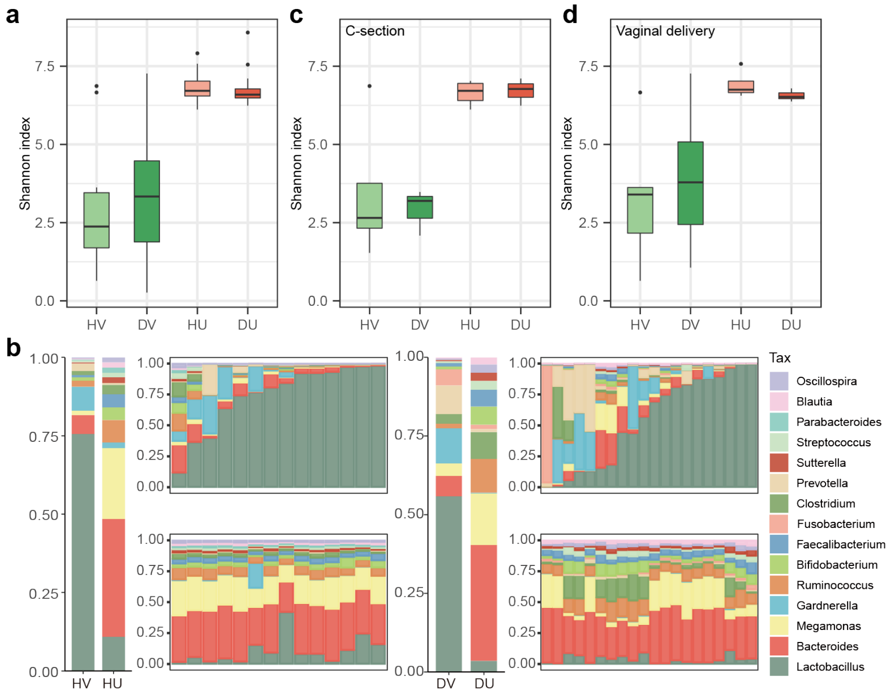

Supplementary Fig. 2 Comparisons between microbiota of chronic endometritis and control when considering age and delivery modes. a Microbial diversity of the uterine and vaginal microbiome in women of similar age (35~45 years old) with different health statuses. **b** Relative abundance of the dominant genera of the uterine and vaginal microbiome in women of similar age (35~45 years old) with different health statuses. The two bars on the left show the average relative abundance of each group. **c-d** Microbial diversities of the uterine and vaginal microbiome in women with different health statuses and delivery modes. HV, DV, HU, and DU represent healthy vagina, diseased vagina, healthy uterus, and diseased uterus, respectively. For a, c and d, box-plot elements are defined as: center line, median; box limits, upper and lower quartiles; whiskers, 1.5× interquartile range; points, outliers.

a

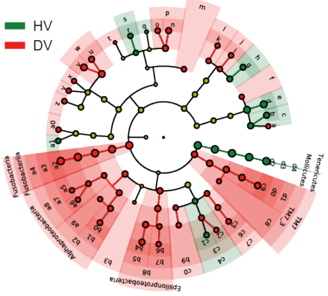

b

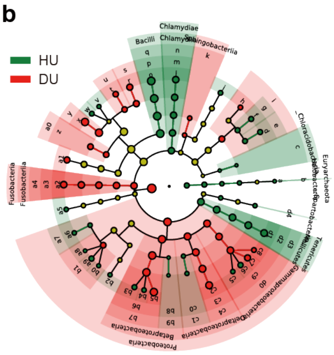

c

Discriminatory bacterial taxa			
Vagina		Uterus	
Health	Disease	Health	Disease
Actinomycetaceae	Alistipes	Ruminococcus	Actinomycetales
Corynebacteriacea	Arcanobacterium	Blastococcus	Clostridiaceae
Corynebacterium	Bacillales	Brevibacteriaceae	Clostridium
Holdemania	Bulleidia	Brevibacterium	Dehalobacteriaceae
Leuconostocaceae	Butyricicoccus	Chlamydia	Dehalobacterium
Mobiluncus	Clostridium	Chlamydiaceae	Frigoribacterium
Parabacteroides	Coprococcus	Chlamydiales	Fusobacteriales
Pediococcus	Enterococcaceae	Citrullus	Leptotrichiaceae
Porphyromonadaceae	Enterococcus	Dysgonomonas	Ruminococcaceae
Weissella	Fusobacteriales	Geodermatophilaceae	Ruminococcus
Acinetobacter	Lachnospiraceae	Halobacteriales	Sneathia
Pseudomonadales	Leptotrichiaceae	Halophilic	Sphingobacteriales
	Loktanella	Lactobacillaceae	Sutterella
	Micrococcaceae	Lactobacillales	Alcaligenaceae
	Mitsuokella	Lactobacillus	Burkholderiales
	Prevotella	Nelumbo	Bilophila
	Prevotellaceae	Oenothera	Desulfovibrionaceae
	Pseudoruegeria	Pelagibacteraceae	Desulfovibrionales
	Rhodobacteraceae	RB41	Klebsiella
	Rhodobacterales	Shuttleworthia	Xenorhabdus
	Rikenellaceae	Victivallis	Enterobacteriaceae
	Shuttleworthia	Sphingobium	Enterobacteriales
	Sneathia	Alcaligenes	
	Rickettsiales	Rhodospirillum	
	Campylobacter	Bacteriovoracaceae	
	Campylobacteraceae	Bdellovibrionales	
	Helicobacter	Citrobacter	
	Helicobacteraceae	Erwinia	
	Campylobacterales	Mycoplasmataceae	
	Succinivibrionaceae	Mycoplasmatales	
	Aeromonadales	Chthoniobacteraceae	
	Moraxella		
	Moraxellaceae		

Supplementary Fig. 3 Linear discriminant analysis (LDA) effect size (LEfSe) and discriminatory bacterial taxa of the uterine and vaginal microbiota of the women suffering from chronic endometritis. a-b HV, DV, HU, and DU represent healthy vagina, diseased vagina, healthy uterus, and diseased uterus, respectively. **c** A value of log LDA score >3.0 was used as a threshold to identify significantly differential signature better discriminating the microbiota of women suffering from chronic endometritis (disease) or health.

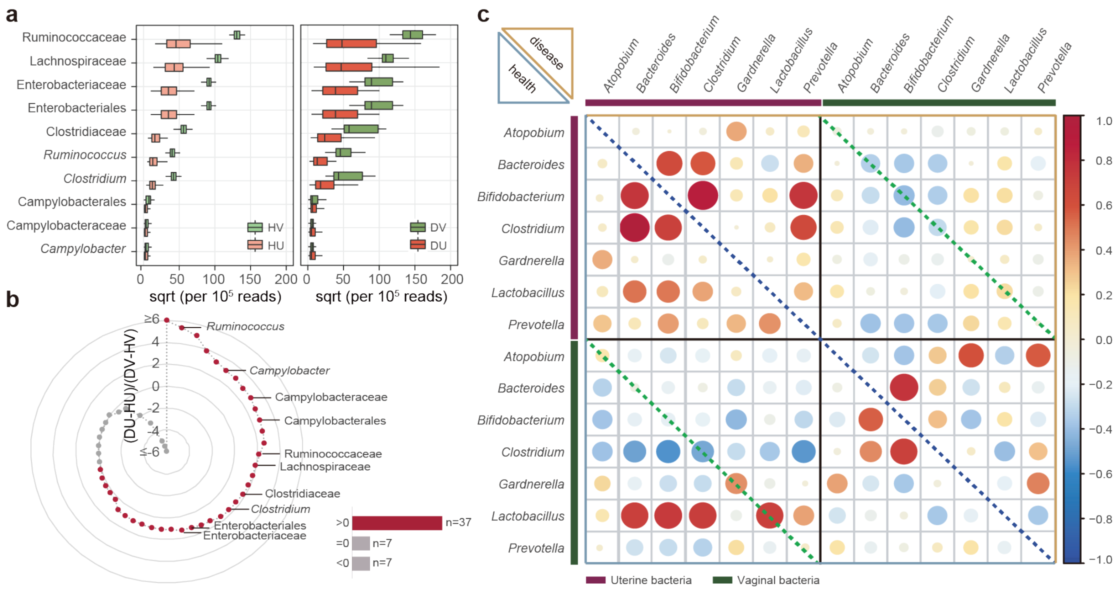

Supplementary Fig. 4 Co-variation and co-occurrence between the uterine and vaginal microbiota associated with chronic endometritis. a Relative abundance and rank of the high abundant discriminatory bacteria. Box-plot elements are defined as: center line, median; box limits, upper and lower quartiles; whiskers, 1.5× interquartile range. **b** Change direction in the relative abundance of the discriminatory bacteria between the health and disease groups. The change direction is measured by the ratio of DU_{median}-HU_{median}/U_{mean} to DV_{median}-HV_{median}/V_{mean} , in which HV, DV, HU, and DU represent the log10 (relative abundance) of certain bacterial taxa in the microbiota of healthy vagina, diseased vagina, healthy uterus, and diseased uterus, respectively. If the ratio is positive, it means that the bacterial abundance changes with chronic endometritis are in the same direction between the uterine cavity and vagina. **c** Co-occurrence of seven bacterial genera across the two body sites. The blue dotted line divides different health states, and the pies on the two green dashed lines show the correlation of the same genus between the uterine and the vaginal microbiota in health or disease group.

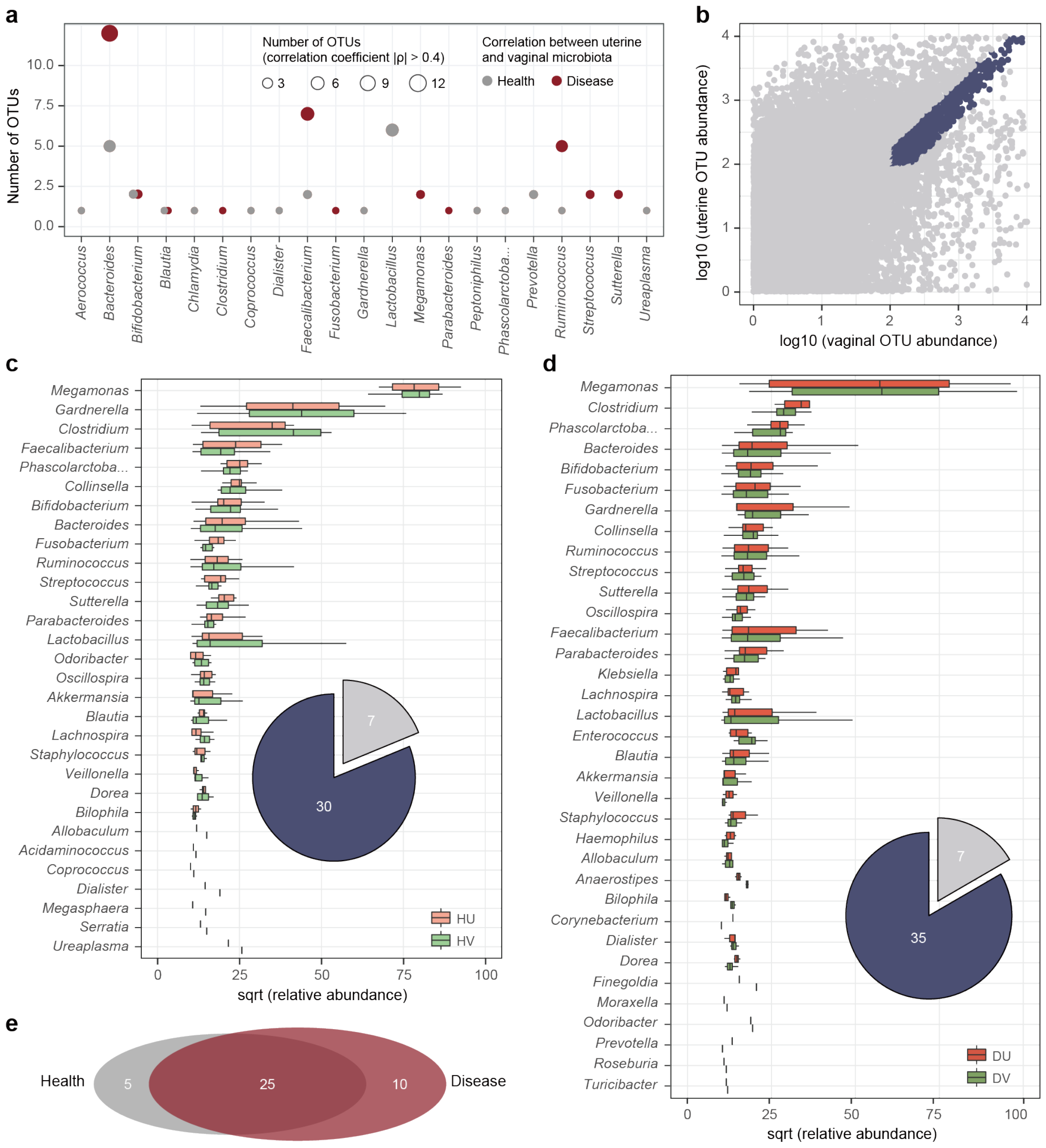

Supplementary Fig. 5 Bacterial genera that tends to covariate with chronic endometritis between the uterine and the vaginal microbiota. a Genera with strong correlation across two body sites. The correlation of each OTU was calculated in healthy and diseased women respectively, and the number of OTUs having correlation coefficient $|\rho| > 0.4$ was counted. **b** OTUs with high and similar abundance in the uterine cavity and vagina (OUVs, dots in dark blue). **c** Taxonomic classification of OUVs and the relative abundance of OUVs genera in healthy women. Dark blue (30/37) and grey (7/37) sectors in pie diagram show the number of OUVs and non-OUVs genera, respectively. **d** Taxonomic classification of OUVs and the relative abundance of OUVs genera in women suffering from chronic endometritis. Dark blue (35/42) and grey (7/42) sectors in pie diagram show the number of OUVs and non-OUVs genera, respectively. **e** Overlap of OUV between health (25/30) and disease (25/35) group. HV, DV, HU, and DU represent healthy vagina, diseased vagina, healthy uterus, and diseased uterus, respectively. For c and d, box-plot elements are defined as: center line, median; box limits, upper and lower quartiles; whiskers, 1.5× interquartile range.

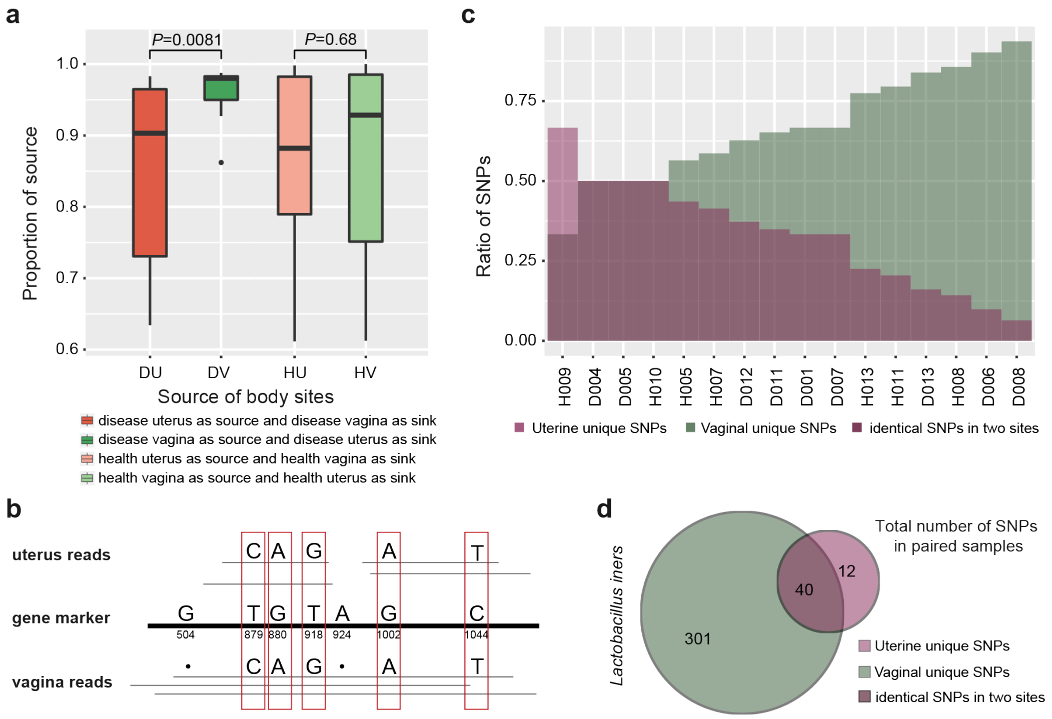

Supplementary Fig. 6 Genus-level and strain-level bacterial source tracking between uterus and vagina. a Source tracking of uterine and vaginal microbiota of the same subject based on 16S rRNA sequencing data. HV, DV, HU, and DU represent healthy vagina, diseased vagina, healthy uterus, and diseased uterus, respectively. Box-plot elements are defined as: center line, median; box limits, upper and lower quartiles; whiskers, 1.5× interquartile range; points, outliers. *P* values were determined by two-tailed Wilcoxon test. **b** Principle of strain-level bacterial source tracking. Uterus-vagina paired samples were aligned to the same species-specific gene marker. Red frames represent identical SNPs shared by each read pair in the same position. Dots in vaginal reads represent there is a gap in the same position of paired uterus reads. **c** Unique or identical SNPs in uterus and vagina of each subject. The height of each bar represents the ratio of SNPs in two body sites. Only subjects have identical SNPs in the same position of uterus-vagina paired samples are shown. **d** Number of SNPs in uterus and vagina for an abundant strain *Lactobacillus iners*.

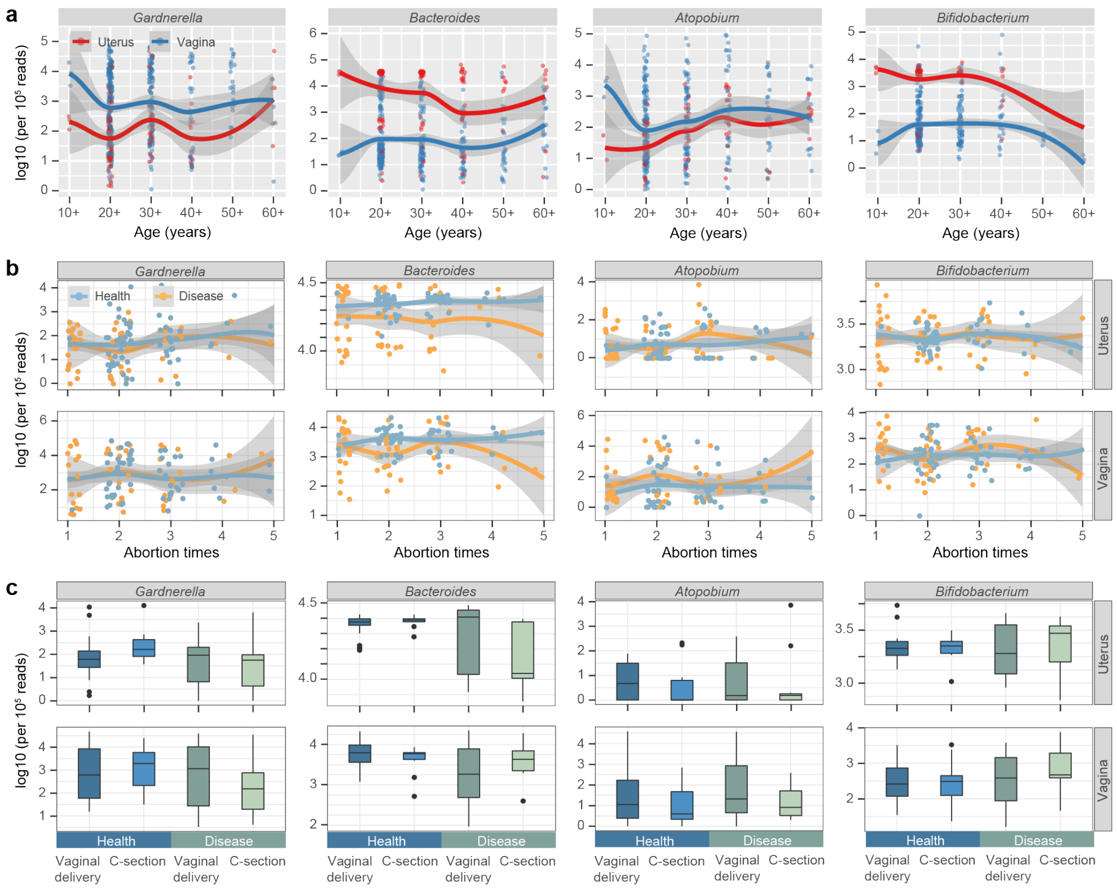

Supplementary Fig. 7 The variation of four bacterial genera in the uterine and vaginal microbiota. a Relative abundance in the uterine (red) and vaginal (blue) microbiota along with women's age. The values were normalized to 10^5 reads in each sample. **b** Relative abundance along with abortion times. For a and b, the shadow around the linear regression trendline shows the 95% confidence interval (CI). **c** Relative abundance along with previous delivery mode of women. Box-plot elements are defined as: center line, median; box limits, upper and lower quartiles; whiskers, 1.5× interquartile range.

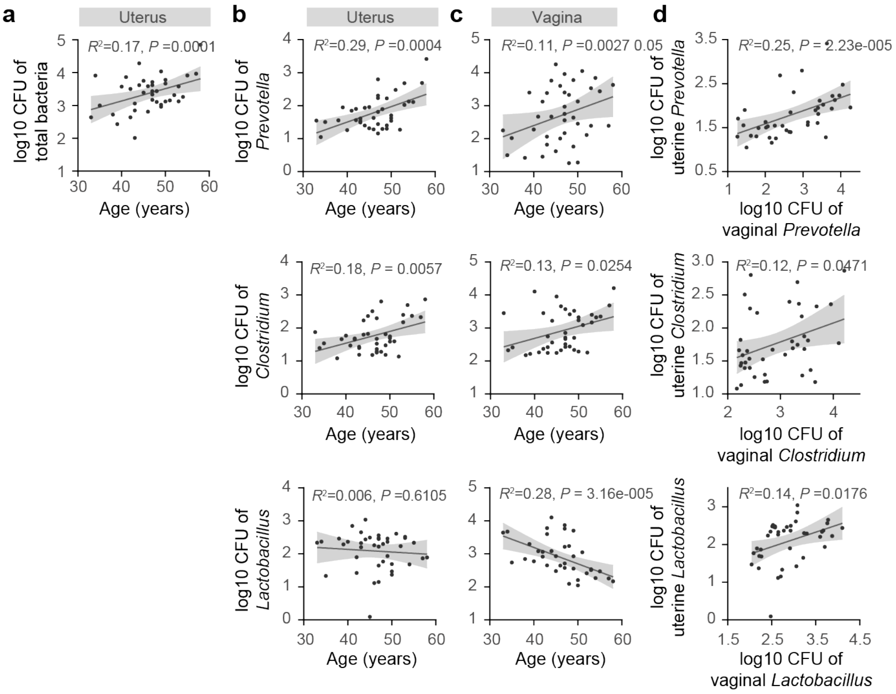

Supplementary Fig. 8 qPCR quantification of the absolute abundance of bacteria in vaginal and uterine flushing fluid. a Biomass of total bacteria at the genus level in uterine samples correlated with the age of women (n = 40). **b-c** Biomass of *Prevotella*, *Clostridium* and *Lactobacillus* in the uterine and the vagina of women with different age, respectively. **d** Correlation of the same bacterial genus between the two body sites. Correlation analyses were performed based on Spearman's rho statistic. For a-d, linear regression was used to analysis the correlation between two pairs of data, the shadow around the trendline shows the 95% confidence interval (CI).

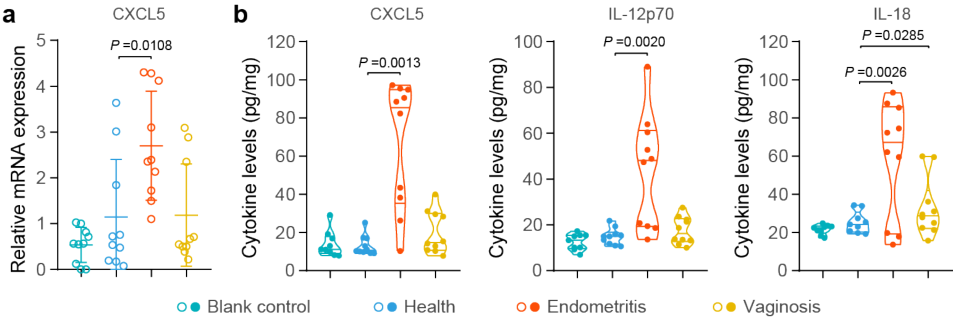

Supplementary Fig. 9 The mRNA expression (a) and cytokine levels of inflammatory factors (b) in the endometrial tissues of the rats transplanting the vaginal microbiota of women to the rats' vagina. Vaginal lavage fluids were collected from 10 women who were healthy, suffering from chronic endometritis or bacterial vaginosis, and then were transplanted into the vagina of SD rats after 1 week of antibiotic treatment. For each group, n=10. *P* values were determined by two-tailed Student's t-test. a, the lines among scatter shows the means ± s.e.m. b, the width of the violin represents the density distribution, and the upper, center and lower lines among points represent upper quartile, median and lower quartile, respectively.

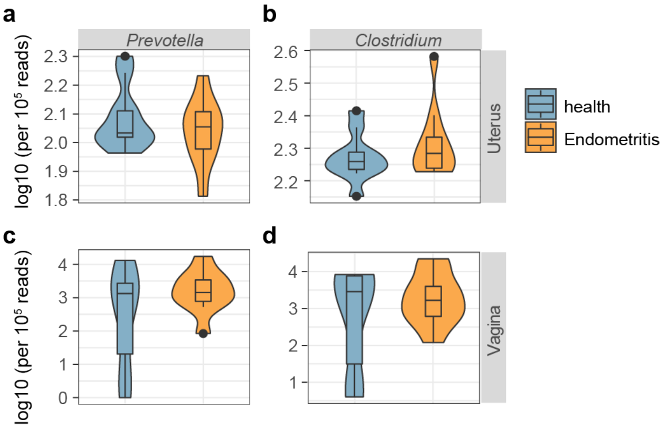

Supplementary Fig. 10 Relative abundance of *Prevotella* and *Clostridum* in the uterine (a-b) and vaginal (c-d) microbiota of healthy women and women suffering from chronic endometritis. Metagenomic sequencing was performed on 10 samples of each group, and reads were respectively mapped to the reference genomes of *Prevotella* and *Clostridum* strains deposited in the Human Microbiome Project (HMP). The violin with box plot shows the median and interquartile range, and the width of the violin represents the density distribution. Box-plot elements are defined as: center line, median; box limits, upper and lower quartiles; whiskers, 1.5× interquartile range; points, outliers.

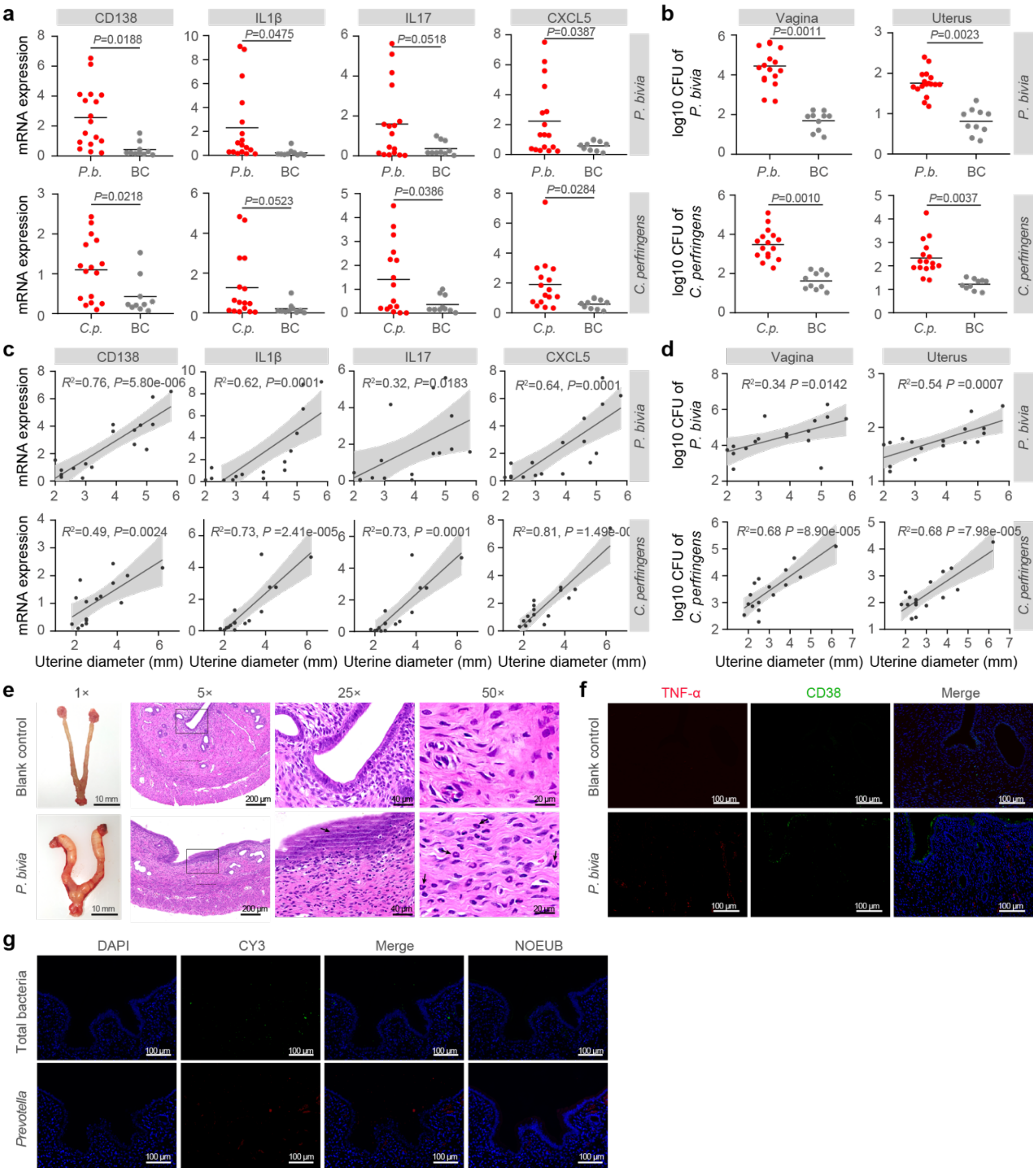

Supplementary Fig. 11 Challenge experiment by injecting *Prevotella bivia* (*P. bivia*, *P.b.*) or *Clostridium perfringens* (*C. perfringens*, *C.p.*) into the vagina of SD rats. a Inflammatory factors of the endometrial tissue in the challenge rats. *P.b.*(n=17), *C.p.* (n=16) and BC rats (n=10). **b** The biomass of the transplanted strain in vagina and uterus quantified by qPCR. *P.b.*(n=17), *C.p.* (n=16) and BC rats (n=10). **c** Correlation between the mRNA expression level of inflammatory factors and uterine diameter. **d** Correlation between the biomass of the transplanted strain in vagina and uterus and uterine diameter. **e** Uterine bodies and hematoxylin-eosin staining of the endometrial tissues in the *P.b.* and BC rats. The 25× and 50× field of view show the areas within the solid and dashed frames of the 5× field of view. The black arrows under 25× field of view show hyperplastic endometrial epithelium. **f** Immunofluorescence assay illustrates the TNF-α and CD38 signals in the endometrial tissue of the *P.b.* and BC rats. **g** Fluorescence *in situ* hybridization (FISH) of total bacteria (green) and *Prevotella* (red)

in the uterine cavity of the *P.b.* rats. For a and b, *P* values were determined by two-tailed Student's t-test, and the bar represents the mean value. For c and d, linear regression was used to analysis the correlation between two pairs of data, and the shadow around the trendline shows the 95% confidence interval (CI). For e-g, images are representative of three independent experiments with similar results.

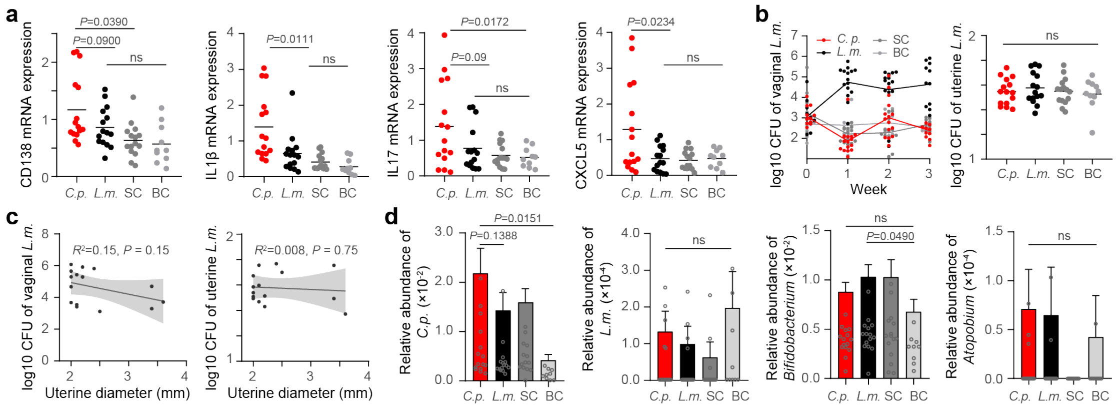

Supplementary Fig. 12 The results of injecting *C.p.*, *Lactobacillus murinus* (*L. murinus*, *L.m.*), supernatant of *C. perfringens* (SC) and PBS (BC) into the vagina of SD rats. a The mRNA expression of inflammatory factors of the endometrial tissue in the vaginal microbiota transplantation (VMT) rats. **b** The biomass of vaginal and uterine *L.m.* in the VMT rats. **c** Correlations of the biomass of vaginal and uterine *L.m.* with uterine diameter. **d** Relative abundance of *C.p.*, *L.m.*, *Bifidobacterium*, and *Atopobium* in the uterine microbiota of the *C.p.* (n=15), *L.m.* (n=15), SC (n=15) and BC (n=10) rat group measured by the 16S rRNA amplicon sequencing. For a, b and d, *P* values were determined by one-way ANOVA with Tukey's multiple comparison post-hoc test, and data are presented as means ± s.e.m; c, linear regression was used to analysis the correlation between two pairs of data, and the shadow around the trendline shows the 95% confidence interval (CI); ns represent non-significant.

Supplementary Tables

Supplementary Table 1 Metadata of the participants recruited in this study.

ID	Age	# pregnancy	# delivery	# abortion	Delivery mode	Diagnosis	Country	Sex
H001	25	1	0	1	-	Health	China	Female
H002	34	2	1	1	-	Health	China	Female
H003	29	2	1	1	-	Health	China	Female
H004	24	1	0	1	-	Health	China	Female
H005	32	1	1	0	-	Health	China	Female
H006	26	0	0	0	-	Health	China	Female
H007	30	2	1	1	-	Health	China	Female
H008	27	1	0	1	-	Health	China	Female
H009	31	1	0	1	-	Health	China	Female
H010	27	2	1	1	-	Health	China	Female
H011	42	2	1	1	-	Health	China	Female
H012	26	2	1	1	-	Health	China	Female
H013	28	2	0	2	-	Health	China	Female
H014	30	2	1	1	-	Health	China	Female
H015	30	3	2	1	Vaginal	Health	China	Female
H016	36	3	1	2	Vaginal	Health	China	Female
H017	24	2	0	2	-	Health	China	Female
H018	32	6	3	3	Vaginal	Health	China	Female
H019	35	1	0	1	-	Health	China	Female

H020	39	3	1	2	Vaginal	Health	China	Female
H021	35	2	1	1	Vaginal	Health	China	Female
H022	33	3	1	2	Vaginal	Health	China	Female
H023	23	1	0	1	-	Health	China	Female
H024	42	3	1	2	Vaginal	Health	China	Female
H025	32	3	1	2	Vaginal	Health	China	Female
H026	27	1	0	1	-	Health	China	Female
H027	28	3	1	2	Csection	Health	China	Female
H028	34	2	1	1	Vaginal	Health	China	Female
H029	29	1	0	1	-	Health	China	Female
H030	26	3	1	2	Csection	Health	China	Female
H031	36	2	1	1	Csection	Health	China	Female
H032	26	1	0	1	-	Health	China	Female
H033	29	3	2	1	Vaginal	Health	China	Female
H034	35	2	1	1	Vaginal	Health	China	Female
H035	20	2	0	2	-	Health	China	Female
H036	30	3	2	1	Csection	Health	China	Female
H037	36	2	0	2	-	Health	China	Female
H038	29	3	1	2	Vaginal	Health	China	Female
H039	37	3	2	1	Vaginal	Health	China	Female
H040	30	1	0	1	-	Health	China	Female
H041	36	3	1	2	Vaginal	Health	China	Female
H042	25	2	0	2	-	Health	China	Female
H043	37	3	1	2	Vaginal	Health	China	Female
H044	29	4	1	3	Vaginal	Health	China	Female

H045	32	4	2	2	Csection	Health	China	Female
H046	33	4	1	3	Vaginal	Health	China	Female
H047	36	2	0	2	-	Health	China	Female
H048	37	3	1	2	Csection	Health	China	Female
H049	25	2	1	1	Csection	Health	China	Female
H050	24	1	0	1	-	Health	China	Female
H051	24	2	1	1	Vaginal	Health	China	Female
H052	25	1	0	1	-	Health	China	Female
H053	26	4	0	4	-	Health	China	Female
H054	22	2	1	1	Vaginal	Health	China	Female
H055	22	1	0	1	-	Health	China	Female
H056	45	4	2	2	Vaginal	Health	China	Female
H057	39	4	1	3	Csection	Health	China	Female
H058	27	2	0	2	-	Health	China	Female
H059	36	4	1	3	Vaginal	Health	China	Female
H060	22	1	0	1	-	Health	China	Female
H061	21	3	0	3	-	Health	China	Female
H062	32	2	1	1	Vaginal	Health	China	Female
H063	24	3	1	2	Csection	Health	China	Female
H064	29	3	1	2	Vaginal	Health	China	Female
H065	25	2	1	1	Vaginal	Health	China	Female
H066	27	2	1	1	Vaginal	Health	China	Female
H067	18	1	0	1	-	Health	China	Female
H068	36	2	2	0	Csection	Health	China	Female
H069	38	3	1	2	Vaginal	Health	China	Female

H070	46	4	1	3	Vaginal	Health	China	Female
H071	19	1	0	1	-	Health	China	Female
H072	32	5	1	4	Csection	Health	China	Female
H073	29	3	2	1	Vaginal	Health	China	Female
D001	26	2	1	1	-	Endometritis	China	Female
D002	51	2	1	1	-	Endometritis	China	Female
D003	53	3	1	2	-	Endometritis	China	Female
D004	25	1	1	0	-	Endometritis	China	Female
D005	46	3	1	2	-	Endometritis	China	Female
D006	47	2	2	0	-	Endometritis	China	Female
D007	48	2	1	1	-	Endometritis	China	Female
D008	32	1	1	0	-	Endometritis	China	Female
D009	34	0	0	0	-	Endometritis	China	Female
D010	43	0	0	0	-	Endometritis	China	Female
D011	42	2	2	0	-	Endometritis	China	Female
D012	33	3	1	2	-	Endometritis	China	Female
D013	-	-	-	0	-	Endometritis	China	Female
D014	-	-	-	0	-	Endometritis	China	Female
D015	58	3	2	1	Vaginal	Endometritis	China	Female
D016	50	2	2	0	Vaginal	Endometritis	China	Female
D017	54	2	1	1	Vaginal	Endometritis	China	Female
D018	47	2	2	0	Vaginal	Endometritis	China	Female
D019	42	4	1	3	Vaginal	Endometritis	China	Female
D020	35	2	1	1	Vaginal	Endometritis	China	Female
D021	47	1	1	0	Vaginal	Endometritis	China	Female

D022	42	2	2	0	Vaginal	Endometritis	China	Female
D023	46	3	1	2	Vaginal	Endometritis	China	Female
D024	50	3	1	2	Vaginal	Endometritis	China	Female
D025	43	4	2	2	Vaginal	Endometritis	China	Female
D026	50	3	1	2	Vaginal	Endometritis	China	Female
D027	43	4	1	3	Csection	Endometritis	China	Female
D028	48	2	2	0	Vaginal	Endometritis	China	Female
D029	57	2	2	0	Vaginal	Endometritis	China	Female
D030	52	3	3	0	Vaginal	Endometritis	China	Female
D031	49	2	2	0	Vaginal	Endometritis	China	Female
D032	45	2	1	1	Vaginal	Endometritis	China	Female
D033	47	2	1	1	Vaginal	Endometritis	China	Female
D034	55	3	3	0	Vaginal	Endometritis	China	Female
D035	53	2	2	0	Vaginal	Endometritis	China	Female
D036	43	1	1	0	Vaginal	Endometritis	China	Female
D037	49	2	2	0	Vaginal	Endometritis	China	Female
D038	39	2	1	1	Vaginal	Endometritis	China	Female
D039	53	3	2	1	Vaginal	Endometritis	China	Female
D040	51	6	2	4	Vaginal	Endometritis	China	Female
D041	48	2	1	1	Vaginal	Endometritis	China	Female
D042	47	3	1	2	Vaginal	Endometritis	China	Female
D043	47	2	1	1	Csection	Endometritis	China	Female
D044	33	3	2	1	Csection	Endometritis	China	Female
D045	46	3	1	2	Csection	Endometritis	China	Female
D046	40	1	1	0	Csection	Endometritis	China	Female

D047	44	1	1	0	Vaginal	Endometritis	China	Female
D048	47	2	1	1	Vaginal	Endometritis	China	Female
D049	34	2	1	1	Vaginal	Endometritis	China	Female
D050	44	3	1	2	Vaginal	Endometritis	China	Female
D051	41	1	1	0	Vaginal	Endometritis	China	Female
D052	45	2	1	1	Vaginal	Endometritis	China	Female
D053	49	1	1	0	Vaginal	Endometritis	China	Female
D054	38	1	1	0	Vaginal	Endometritis	China	Female
D055	52	2	1	1	Csection	Endometritis	China	Female
D056	40	1	1	0	Csection	Endometritis	China	Female
D057	52	2	2	0	Vaginal	Endometritis	China	Female
D058	49	0	0	0	-	Endometritis	China	Female
D059	39	1	1	0	Vaginal	Endometritis	China	Female
D060	64	4	4	0	Vaginal	Endometritis	China	Female
D061	45	3	1	2	Csection	Endometritis	China	Female
D062	69	3	2	1	Vaginal	Endometritis	China	Female
D063	55	1	1	0	Csection	Endometritis	China	Female
D064	42	1	1	0	Vaginal	Endometritis	China	Female
D065	71	5	3	2	Vaginal	Endometritis	China	Female
D066	50	3	2	1	Vaginal	Endometritis	China	Female
D067	49	1	1	0	Vaginal	Endometritis	China	Female
D068	-	-	-	0	-	Endometritis	China	Female
D069	60	1	1	0	Csection	Endometritis	China	Female
D070	55	1	1	0	Csection	Endometritis	China	Female
D071	57	5	2	3	Csection	Endometritis	China	Female

D072	58	3	1	2	Vaginal	Endometritis	China	Female

Supplementary Table 2 Medical records of 1,612 women used to analysis correlation between endometritis with clinical factors.

Individual	Age	# pregnancy	# delivery	# abortion	Diagnosis	Hospital	Country	Sex
D1	27	1	0	0	Endometritis	Hospital 1	China	Female
D2	38	3	1	2	Endometritis	Hospital 1	China	Female
D3	38	2	0	2	Endometritis	Hospital 1	China	Female
D4	31	5	0	4	Endometritis	Hospital 1	China	Female
D5	35	1	0	1	Endometritis	Hospital 1	China	Female
D6	38	3	1	1	Endometritis	Hospital 1	China	Female
D7	36	1	0	1	Endometritis	Hospital 1	China	Female
D8	28	2	0	2	Endometritis	Hospital 1	China	Female
D9	35	3	1	1	Endometritis	Hospital 1	China	Female
D10	35	2	1	0	Endometritis	Hospital 1	China	Female
D11	27	2	0	1	Endometritis	Hospital 1	China	Female
D12	35	3	0	2	Endometritis	Hospital 1	China	Female
D13	37	1	0	0	Endometritis	Hospital 1	China	Female
D14	37	3	0	2	Endometritis	Hospital 1	China	Female
D15	37	1	0	0	Endometritis	Hospital 1	China	Female
D16	36	2	0	2	Endometritis	Hospital 1	China	Female
D17	37	1	1	0	Endometritis	Hospital 1	China	Female
D18	32	2	1	1	Endometritis	Hospital 1	China	Female
D19	37	2	0	1	Endometritis	Hospital 1	China	Female
D20	34	2	1	1	Endometritis	Hospital 1	China	Female
D21	35	1	0	0	Endometritis	Hospital 1	China	Female

D22	37	4	2	1	Endometritis	Hospital 1	China	Female
D23	32	1	0	0	Endometritis	Hospital 1	China	Female
D24	34	3	0	2	Endometritis	Hospital 1	China	Female
D25	37	1	1	0	Endometritis	Hospital 1	China	Female
D26	35	1	0	0	Endometritis	Hospital 1	China	Female
D27	37	2	0	1	Endometritis	Hospital 1	China	Female
D28	35	5	0	2	Endometritis	Hospital 1	China	Female
D29	37	3	0	1	Endometritis	Hospital 1	China	Female
D30	37	1	0	0	Endometritis	Hospital 1	China	Female
D31	33	1	0	0	Endometritis	Hospital 1	China	Female
D32	37	1	0	1	Endometritis	Hospital 1	China	Female
D33	36	1	0	0	Endometritis	Hospital 1	China	Female
D34	34	2	1	1	Endometritis	Hospital 1	China	Female
D35	39	2	2	0	Endometritis	Hospital 1	China	Female
D36	36	1	0	0	Endometritis	Hospital 1	China	Female
D37	36	3	0	2	Endometritis	Hospital 1	China	Female
D38	37	1	0	0	Endometritis	Hospital 1	China	Female
D39	35	4	0	2	Endometritis	Hospital 1	China	Female
D40	35	1	0	0	Endometritis	Hospital 1	China	Female
D41	35	3	1	2	Endometritis	Hospital 1	China	Female
D42	33	2	0	1	Endometritis	Hospital 1	China	Female
D43	34	1	1	0	Endometritis	Hospital 1	China	Female
D44	36	1	1	0	Endometritis	Hospital 1	China	Female
D45	37	1	0	0	Endometritis	Hospital 1	China	Female
D46	33	3	0	0	Endometritis	Hospital 1	China	Female

D47	35	2	0	1	Endometritis	Hospital 1	China	Female
D48	33	1	0	0	Endometritis	Hospital 1	China	Female
D49	37	1	0	0	Endometritis	Hospital 1	China	Female
D50	35	2	0	2	Endometritis	Hospital 1	China	Female
D51	35	4	1	2	Endometritis	Hospital 1	China	Female
D52	33	1	0	0	Endometritis	Hospital 1	China	Female
D53	37	1	0	0	Endometritis	Hospital 1	China	Female
D54	32	2	0	2	Endometritis	Hospital 1	China	Female
D55	37	4	0	3	Endometritis	Hospital 1	China	Female
D56	34	2	1	1	Endometritis	Hospital 1	China	Female
D57	33	3	1	2	Endometritis	Hospital 1	China	Female
D58	35	1	0	0	Endometritis	Hospital 1	China	Female
D59	26	1	0	0	Endometritis	Hospital 1	China	Female
D60	36	1	0	0	Endometritis	Hospital 1	China	Female
D61	36	6	1	4	Endometritis	Hospital 1	China	Female
D62	37	2	0	1	Endometritis	Hospital 1	China	Female
D63	36	1	1	0	Endometritis	Hospital 1	China	Female
D64	38	2	0	0	Endometritis	Hospital 1	China	Female
D65	36	1	0	0	Endometritis	Hospital 1	China	Female
D66	36	1	0	0	Endometritis	Hospital 1	China	Female
D67	36	1	0	0	Endometritis	Hospital 1	China	Female
D68	37	2	1	0	Endometritis	Hospital 1	China	Female
D69	33	1	0	0	Endometritis	Hospital 1	China	Female
D70	36	2	0	2	Endometritis	Hospital 1	China	Female
D71	37	4	0	2	Endometritis	Hospital 1	China	Female

D72	26	2	0	1	Endometritis	Hospital 1	China	Female
D73	33	1	0	0	Endometritis	Hospital 1	China	Female
D74	37	3	1	1	Endometritis	Hospital 1	China	Female
D75	37	2	0	1	Endometritis	Hospital 1	China	Female
D76	37	1	0	0	Endometritis	Hospital 1	China	Female
D77	36	3	1	2	Endometritis	Hospital 1	China	Female
D78	37	1	0	0	Endometritis	Hospital 1	China	Female
D79	37	2	0	1	Endometritis	Hospital 1	China	Female
D80	36	1	0	0	Endometritis	Hospital 1	China	Female
D81	35	2	1	0	Endometritis	Hospital 1	China	Female
D82	37	5	2	2	Endometritis	Hospital 1	China	Female
D83	30	2	0	0	Endometritis	Hospital 1	China	Female
D84	37	2	1	1	Endometritis	Hospital 1	China	Female
D85	33	1	0	0	Endometritis	Hospital 1	China	Female
D86	36	1	1	0	Endometritis	Hospital 1	China	Female
D87	34	4	1	2	Endometritis	Hospital 1	China	Female
D88	35	2	1	0	Endometritis	Hospital 1	China	Female
D89	37	4	0	3	Endometritis	Hospital 1	China	Female
D90	34	1	0	0	Endometritis	Hospital 1	China	Female
D91	33	3	0	0	Endometritis	Hospital 1	China	Female
D92	38	1	0	0	Endometritis	Hospital 1	China	Female
D93	37	1	0	0	Endometritis	Hospital 1	China	Female
D94	36	2	0	1	Endometritis	Hospital 1	China	Female
D95	34	2	0	1	Endometritis	Hospital 1	China	Female
D96	37	1	0	0	Endometritis	Hospital 1	China	Female

D97	35	2	0	1	Endometritis	Hospital 1	China	Female
D98	36	1	1	0	Endometritis	Hospital 1	China	Female
D99	37	1	0	0	Endometritis	Hospital 1	China	Female
D100	35	3	1	0	Endometritis	Hospital 1	China	Female
D101	34	1	0	0	Endometritis	Hospital 1	China	Female
D102	34	1	0	0	Endometritis	Hospital 1	China	Female
D103	37	1	1	0	Endometritis	Hospital 1	China	Female
D104	34	2	1	1	Endometritis	Hospital 1	China	Female
D105	35	3	2	1	Endometritis	Hospital 1	China	Female
D106	37	1	0	0	Endometritis	Hospital 1	China	Female
D107	35	1	0	0	Endometritis	Hospital 1	China	Female
D108	36	1	0	0	Endometritis	Hospital 1	China	Female
D109	32	2	1	0	Endometritis	Hospital 1	China	Female
D110	36	2	0	1	Endometritis	Hospital 1	China	Female
D111	37	2	0	0	Endometritis	Hospital 1	China	Female
D112	35	1	0	0	Endometritis	Hospital 1	China	Female
D113	36	1	0	0	Endometritis	Hospital 1	China	Female
D114	35	2	0	0	Endometritis	Hospital 1	China	Female
D115	34	5	1	1	Endometritis	Hospital 1	China	Female
D116	37	1	1	0	Endometritis	Hospital 1	China	Female
D117	36	2	1	0	Endometritis	Hospital 1	China	Female
D118	35	3	0	1	Endometritis	Hospital 1	China	Female
D119	37	2	0	1	Endometritis	Hospital 1	China	Female
D120	35	1	0	0	Endometritis	Hospital 1	China	Female
D121	34	2	1	0	Endometritis	Hospital 1	China	Female

D122	37	1	1	0	Endometritis	Hospital 1	China	Female
D123	37	1	0	1	Endometritis	Hospital 1	China	Female
D124	29	2	1	1	Endometritis	Hospital 1	China	Female
D125	37	4	1	3	Endometritis	Hospital 1	China	Female
D126	35	1	0	1	Endometritis	Hospital 1	China	Female
D127	37	1	1	0	Endometritis	Hospital 1	China	Female
D128	37	2	0	1	Endometritis	Hospital 1	China	Female
D129	32	1	0	0	Endometritis	Hospital 1	China	Female
D130	36	2	0	2	Endometritis	Hospital 1	China	Female
D131	37	3	0	2	Endometritis	Hospital 1	China	Female
D132	37	2	0	1	Endometritis	Hospital 1	China	Female
D133	36	4	1	2	Endometritis	Hospital 1	China	Female
D134	37	1	0	0	Endometritis	Hospital 1	China	Female
D135	36	6	0	4	Endometritis	Hospital 1	China	Female
D136	37	1	0	0	Endometritis	Hospital 1	China	Female
D137	35	1	0	0	Endometritis	Hospital 1	China	Female
D138	38	1	0	0	Endometritis	Hospital 1	China	Female
D139	37	2	2	0	Endometritis	Hospital 1	China	Female
D140	36	1	0	0	Endometritis	Hospital 1	China	Female
D141	38	1	1	0	Endometritis	Hospital 1	China	Female
D142	33	2	2	0	Endometritis	Hospital 1	China	Female
D143	35	1	0	0	Endometritis	Hospital 1	China	Female
D144	37	1	1	0	Endometritis	Hospital 1	China	Female
D145	36	1	1	0	Endometritis	Hospital 1	China	Female
D146	35	1	0	0	Endometritis	Hospital 1	China	Female

D147	34	2	0	2	Endometritis	Hospital 1	China	Female
D148	37	1	1	0	Endometritis	Hospital 1	China	Female
D149	37	1	0	0	Endometritis	Hospital 1	China	Female
D150	37	1	0	0	Endometritis	Hospital 1	China	Female
D151	33	1	0	0	Endometritis	Hospital 1	China	Female
D152	37	3	0	2	Endometritis	Hospital 1	China	Female
D153	38	1	0	0	Endometritis	Hospital 1	China	Female
D154	36	1	0	0	Endometritis	Hospital 1	China	Female
D155	34	2	1	0	Endometritis	Hospital 1	China	Female
D156	37	1	1	0	Endometritis	Hospital 1	China	Female
D157	36	1	0	0	Endometritis	Hospital 1	China	Female
D158	37	1	0	0	Endometritis	Hospital 1	China	Female
D159	37	4	0	3	Endometritis	Hospital 1	China	Female
D160	33	1	0	0	Endometritis	Hospital 1	China	Female
D161	35	6	1	4	Endometritis	Hospital 1	China	Female
D162	35	3	2	1	Endometritis	Hospital 1	China	Female
D163	33	1	0	0	Endometritis	Hospital 1	China	Female
D164	35	2	0	1	Endometritis	Hospital 1	China	Female
D165	37	1	0	0	Endometritis	Hospital 1	China	Female
D166	36	3	0	3	Endometritis	Hospital 1	China	Female
D167	37	2	1	1	Endometritis	Hospital 1	China	Female
D168	37	1	0	0	Endometritis	Hospital 1	China	Female
D169	32	1	0	0	Endometritis	Hospital 1	China	Female
D170	36	2	0	2	Endometritis	Hospital 1	China	Female
D171	36	2	0	1	Endometritis	Hospital 1	China	Female

D172	36	3	1	0	Endometritis	Hospital 1	China	Female
D173	28	2	0	0	Endometritis	Hospital 1	China	Female
D174	37	1	0	0	Endometritis	Hospital 1	China	Female
D175	37	1	1	0	Endometritis	Hospital 1	China	Female
D176	36	1	0	0	Endometritis	Hospital 1	China	Female
D177	37	1	1	0	Endometritis	Hospital 1	China	Female
D178	37	2	0	1	Endometritis	Hospital 1	China	Female
D179	37	1	0	0	Endometritis	Hospital 1	China	Female
D180	34	1	0	0	Endometritis	Hospital 1	China	Female
D181	37	4	0	2	Endometritis	Hospital 1	China	Female
D182	36	1	0	0	Endometritis	Hospital 1	China	Female
D183	33	3	0	0	Endometritis	Hospital 1	China	Female
D184	32	1	0	0	Endometritis	Hospital 1	China	Female
D185	36	1	0	0	Endometritis	Hospital 1	China	Female
D186	36	2	1	0	Endometritis	Hospital 1	China	Female
D187	37	1	0	0	Endometritis	Hospital 1	China	Female
D188	35	1	1	0	Endometritis	Hospital 1	China	Female
D189	36	3	1	0	Endometritis	Hospital 1	China	Female
D190	35	1	0	0	Endometritis	Hospital 1	China	Female
D191	35	1	1	0	Endometritis	Hospital 1	China	Female
D192	38	2	1	1	Endometritis	Hospital 1	China	Female
D193	37	2	0	1	Endometritis	Hospital 1	China	Female
D194	37	1	0	0	Endometritis	Hospital 1	China	Female
D195	37	5	1	4	Endometritis	Hospital 1	China	Female
D196	36	3	0	1	Endometritis	Hospital 1	China	Female

D197	35	2	0	1	Endometritis	Hospital 1	China	Female
D198	37	1	0	0	Endometritis	Hospital 1	China	Female
D199	37	1	0	0	Endometritis	Hospital 1	China	Female
D200	37	2	0	1	Endometritis	Hospital 1	China	Female
D201	37	1	0	0	Endometritis	Hospital 1	China	Female
D202	37	1	0	0	Endometritis	Hospital 1	China	Female
D203	37	4	0	3	Endometritis	Hospital 1	China	Female
D204	35	1	0	0	Endometritis	Hospital 1	China	Female
D205	34	2	0	0	Endometritis	Hospital 1	China	Female
D206	35	1	0	0	Endometritis	Hospital 1	China	Female
D207	32	3	1	2	Endometritis	Hospital 1	China	Female
D208	37	4	0	3	Endometritis	Hospital 1	China	Female
D209	39	2	0	1	Endometritis	Hospital 1	China	Female
D210	30	1	0	0	Endometritis	Hospital 1	China	Female
D211	37	1	0	0	Endometritis	Hospital 1	China	Female
D212	37	1	1	0	Endometritis	Hospital 1	China	Female
D213	37	2	1	1	Endometritis	Hospital 1	China	Female
D214	37	1	1	0	Endometritis	Hospital 1	China	Female
D215	33	3	0	2	Endometritis	Hospital 1	China	Female
D216	37	1	0	0	Endometritis	Hospital 1	China	Female
D217	37	1	0	0	Endometritis	Hospital 1	China	Female
D218	37	1	0	0	Endometritis	Hospital 1	China	Female
D219	37	6	0	4	Endometritis	Hospital 1	China	Female
D220	35	1	1	0	Endometritis	Hospital 1	China	Female
D221	31	1	0	0	Endometritis	Hospital 1	China	Female

D222	35	4	0	3	Endometritis	Hospital 1	China	Female
D223	29	1	0	0	Endometritis	Hospital 1	China	Female
D224	36	2	0	2	Endometritis	Hospital 1	China	Female
D225	32	2	2	0	Endometritis	Hospital 1	China	Female
D226	33	1	1	0	Endometritis	Hospital 1	China	Female
D227	29	3	0	1	Endometritis	Hospital 1	China	Female
D228	35	4	1	2	Endometritis	Hospital 1	China	Female
D229	34	2	0	0	Endometritis	Hospital 1	China	Female
D230	37	2	0	0	Endometritis	Hospital 1	China	Female
D231	37	1	0	0	Endometritis	Hospital 1	China	Female
D232	30	1	0	0	Endometritis	Hospital 1	China	Female
D233	33	1	0	0	Endometritis	Hospital 1	China	Female
D234	36	2	0	1	Endometritis	Hospital 1	China	Female
D235	32	1	1	0	Endometritis	Hospital 1	China	Female
D236	36	5	1	3	Endometritis	Hospital 1	China	Female
D237	37	2	0	1	Endometritis	Hospital 1	China	Female
D238	34	4	0	2	Endometritis	Hospital 1	China	Female
D239	34	1	0	0	Endometritis	Hospital 1	China	Female
D240	38	3	0	0	Endometritis	Hospital 1	China	Female
D241	36	1	1	0	Endometritis	Hospital 1	China	Female
D242	37	2	0	0	Endometritis	Hospital 1	China	Female
D243	36	1	1	0	Endometritis	Hospital 1	China	Female
D244	37	2	0	1	Endometritis	Hospital 1	China	Female
D245	37	1	0	0	Endometritis	Hospital 1	China	Female
D246	33	2	0	0	Endometritis	Hospital 1	China	Female

D247	32	6	0	4	Endometritis	Hospital 1	China	Female
D248	37	1	1	0	Endometritis	Hospital 1	China	Female
D249	34	3	1	2	Endometritis	Hospital 1	China	Female
D250	37	3	0	1	Endometritis	Hospital 1	China	Female
D251	35	1	1	0	Endometritis	Hospital 1	China	Female
D252	34	2	2	0	Endometritis	Hospital 1	China	Female
D253	35	1	0	0	Endometritis	Hospital 1	China	Female
D254	37	2	0	0	Endometritis	Hospital 1	China	Female
D255	35	2	0	1	Endometritis	Hospital 1	China	Female
D256	38	5	1	4	Endometritis	Hospital 1	China	Female
D257	38	1	0	0	Endometritis	Hospital 1	China	Female
D258	33	4	0	2	Endometritis	Hospital 1	China	Female
D259	37	2	0	0	Endometritis	Hospital 1	China	Female
D260	34	1	1	0	Endometritis	Hospital 1	China	Female
D261	36	1	0	0	Endometritis	Hospital 1	China	Female
D262	35	1	1	0	Endometritis	Hospital 1	China	Female
D263	37	1	1	0	Endometritis	Hospital 1	China	Female
D264	37	1	0	0	Endometritis	Hospital 1	China	Female
D265	31	2	0	0	Endometritis	Hospital 1	China	Female
D266	37	1	0	0	Endometritis	Hospital 1	China	Female
D267	37	1	0	0	Endometritis	Hospital 1	China	Female
D268	34	1	1	0	Endometritis	Hospital 1	China	Female
D269	33	2	1	0	Endometritis	Hospital 1	China	Female
D270	38	3	0	0	Endometritis	Hospital 1	China	Female
D271	35	3	0	2	Endometritis	Hospital 1	China	Female

D272	36	1	1	0	Endometritis	Hospital 1	China	Female
D273	36	1	1	0	Endometritis	Hospital 1	China	Female
D274	37	2	0	1	Endometritis	Hospital 1	China	Female
D275	35	2	2	0	Endometritis	Hospital 1	China	Female
D276	37	1	0	0	Endometritis	Hospital 1	China	Female
D277	38	3	1	0	Endometritis	Hospital 1	China	Female
D278	37	2	0	1	Endometritis	Hospital 1	China	Female
D279	36	1	1	0	Endometritis	Hospital 1	China	Female
D280	35	1	1	0	Endometritis	Hospital 1	China	Female
D281	37	3	0	2	Endometritis	Hospital 1	China	Female
D282	36	2	1	1	Endometritis	Hospital 1	China	Female
D283	36	1	0	0	Endometritis	Hospital 1	China	Female
D284	36	3	0	0	Endometritis	Hospital 1	China	Female
D285	37	1	1	0	Endometritis	Hospital 1	China	Female
D286	37	1	1	0	Endometritis	Hospital 1	China	Female
D287	35	1	0	0	Endometritis	Hospital 1	China	Female
D288	36	1	1	0	Endometritis	Hospital 1	China	Female
D289	38	2	1	0	Endometritis	Hospital 1	China	Female
D290	31	2	0	1	Endometritis	Hospital 1	China	Female
D291	31	1	0	0	Endometritis	Hospital 1	China	Female
D292	37	3	0	2	Endometritis	Hospital 1	China	Female
D293	34	2	0	0	Endometritis	Hospital 1	China	Female
D294	37	4	1	3	Endometritis	Hospital 1	China	Female
D295	32	2	0	1	Endometritis	Hospital 1	China	Female
D296	37	1	0	0	Endometritis	Hospital 1	China	Female

D297	38	4	0	3	Endometritis	Hospital 1	China	Female
D298	38	3	0	2	Endometritis	Hospital 1	China	Female
D299	37	1	0	0	Endometritis	Hospital 1	China	Female
D300	31	1	0	0	Endometritis	Hospital 1	China	Female
D301	36	3	2	1	Endometritis	Hospital 1	China	Female
D302	38	2	0	0	Endometritis	Hospital 1	China	Female
D303	37	1	0	0	Endometritis	Hospital 1	China	Female
D304	37	3	2	2	Endometritis	Hospital 1	China	Female
D305	38	2	1	1	Endometritis	Hospital 1	China	Female
D306	38	1	0	0	Endometritis	Hospital 1	China	Female
D307	33	1	0	0	Endometritis	Hospital 1	China	Female
D308	37	2	1	1	Endometritis	Hospital 1	China	Female
D309	37	1	1	0	Endometritis	Hospital 1	China	Female
D310	32	1	0	0	Endometritis	Hospital 1	China	Female
D311	26	3	0	2	Endometritis	Hospital 1	China	Female
D312	37	2	1	1	Endometritis	Hospital 1	China	Female
D313	34	1	0	0	Endometritis	Hospital 1	China	Female
D314	36	1	1	0	Endometritis	Hospital 1	China	Female
D315	37	1	0	0	Endometritis	Hospital 1	China	Female
D316	37	1	0	0	Endometritis	Hospital 1	China	Female
D317	37	2	0	2	Endometritis	Hospital 1	China	Female
D318	36	1	0	0	Endometritis	Hospital 1	China	Female
D319	36	2	0	0	Endometritis	Hospital 1	China	Female
D320	27	6	0	4	Endometritis	Hospital 1	China	Female
D321	35	2	0	1	Endometritis	Hospital 1	China	Female

D322	31	1	1	0	Endometritis	Hospital 1	China	Female
D323	37	2	0	1	Endometritis	Hospital 1	China	Female
D324	37	1	1	0	Endometritis	Hospital 1	China	Female
D325	35	2	0	1	Endometritis	Hospital 1	China	Female
D326	29	1	1	0	Endometritis	Hospital 1	China	Female
D327	37	1	1	0	Endometritis	Hospital 1	China	Female
D328	37	1	1	0	Endometritis	Hospital 1	China	Female
D329	39	1	0	0	Endometritis	Hospital 1	China	Female
D330	37	1	0	0	Endometritis	Hospital 1	China	Female
D331	32	3	0	1	Endometritis	Hospital 1	China	Female
D332	36	2	0	1	Endometritis	Hospital 1	China	Female
D333	30	1	1	0	Endometritis	Hospital 1	China	Female
D334	36	1	0	0	Endometritis	Hospital 1	China	Female
D335	35	2	0	1	Endometritis	Hospital 1	China	Female
D336	37	2	0	1	Endometritis	Hospital 1	China	Female
D337	36	1	0	0	Endometritis	Hospital 1	China	Female
D338	36	3	1	2	Endometritis	Hospital 1	China	Female
D339	37	2	1	1	Endometritis	Hospital 1	China	Female
D340	31	1	1	0	Endometritis	Hospital 1	China	Female
D341	37	3	2	1	Endometritis	Hospital 1	China	Female
D342	34	1	1	0	Endometritis	Hospital 1	China	Female
D343	35	1	0	0	Endometritis	Hospital 1	China	Female
D344	32	2	0	1	Endometritis	Hospital 1	China	Female
D345	37	2	1	1	Endometritis	Hospital 1	China	Female
D346	35	1	1	0	Endometritis	Hospital 1	China	Female

D347	36	2	1	1	Endometritis	Hospital 1	China	Female
D348	35	1	0	0	Endometritis	Hospital 1	China	Female
D349	31	1	0	1	Endometritis	Hospital 1	China	Female
D350	37	2	0	0	Endometritis	Hospital 1	China	Female
D351	35	2	0	2	Endometritis	Hospital 1	China	Female
D352	36	3	0	3	Endometritis	Hospital 1	China	Female
D353	36	2	0	1	Endometritis	Hospital 1	China	Female
D354	37	1	0	1	Endometritis	Hospital 1	China	Female
D355	37	2	1	1	Endometritis	Hospital 1	China	Female
D356	32	6	1	4	Endometritis	Hospital 1	China	Female
D357	37	1	0	0	Endometritis	Hospital 1	China	Female
D358	36	1	1	0	Endometritis	Hospital 1	China	Female
D359	37	1	0	0	Endometritis	Hospital 1	China	Female
D360	33	1	0	1	Endometritis	Hospital 1	China	Female
D361	35	3	1	2	Endometritis	Hospital 1	China	Female
D362	37	2	0	1	Endometritis	Hospital 1	China	Female
D363	36	1	0	1	Endometritis	Hospital 1	China	Female
D364	33	5	1	3	Endometritis	Hospital 1	China	Female
D365	37	1	0	0	Endometritis	Hospital 1	China	Female
D366	35	2	0	1	Endometritis	Hospital 1	China	Female
D367	38	1	0	0	Endometritis	Hospital 1	China	Female
D368	38	2	0	1	Endometritis	Hospital 1	China	Female
D369	37	2	0	0	Endometritis	Hospital 1	China	Female
D370	39	2	0	1	Endometritis	Hospital 1	China	Female
D371	35	2	0	0	Endometritis	Hospital 1	China	Female

D372	39	2	0	2	Endometritis	Hospital 1	China	Female
D373	37	2	0	1	Endometritis	Hospital 1	China	Female
D374	34	1	0	0	Endometritis	Hospital 1	China	Female
D375	36	1	0	0	Endometritis	Hospital 1	China	Female
D376	35	1	0	1	Endometritis	Hospital 1	China	Female
D377	37	1	0	0	Endometritis	Hospital 1	China	Female
D378	34	2	0	1	Endometritis	Hospital 1	China	Female
D379	36	3	1	1	Endometritis	Hospital 1	China	Female
D380	35	3	1	1	Endometritis	Hospital 1	China	Female
D381	36	1	0	0	Endometritis	Hospital 1	China	Female
D382	37	2	0	0	Endometritis	Hospital 1	China	Female
D383	37	5	1	3	Endometritis	Hospital 1	China	Female
D384	35	1	0	0	Endometritis	Hospital 1	China	Female
D385	36	1	0	0	Endometritis	Hospital 1	China	Female
D386	36	3	0	2	Endometritis	Hospital 1	China	Female
D387	38	1	0	0	Endometritis	Hospital 1	China	Female
D388	38	2	0	2	Endometritis	Hospital 1	China	Female
D389	32	1	0	0	Endometritis	Hospital 1	China	Female
D390	35	1	0	0	Endometritis	Hospital 1	China	Female
D391	38	3	0	2	Endometritis	Hospital 1	China	Female
D392	33	1	0	0	Endometritis	Hospital 1	China	Female
D393	38	3	1	1	Endometritis	Hospital 1	China	Female
D394	31	2	0	2	Endometritis	Hospital 1	China	Female
D395	33	1	0	0	Endometritis	Hospital 1	China	Female
D396	35	3	0	3	Endometritis	Hospital 1	China	Female

D397	30	1	0	0	Endometritis	Hospital 1	China	Female
D398	37	2	0	1	Endometritis	Hospital 1	China	Female
D399	34	1	0	1	Endometritis	Hospital 1	China	Female
D400	34	1	0	0	Endometritis	Hospital 1	China	Female
D401	36	2	0	1	Endometritis	Hospital 1	China	Female
D402	37	1	0	0	Endometritis	Hospital 1	China	Female
D403	37	2	0	0	Endometritis	Hospital 1	China	Female
D404	35	1	0	0	Endometritis	Hospital 1	China	Female
D405	37	1	0	0	Endometritis	Hospital 1	China	Female
D406	37	1	0	0	Endometritis	Hospital 1	China	Female
D407	36	1	0	0	Endometritis	Hospital 1	China	Female
D408	36	5	0	4	Endometritis	Hospital 1	China	Female
D409	30	3	1	2	Endometritis	Hospital 1	China	Female
D410	37	2	0	1	Endometritis	Hospital 1	China	Female
D411	35	3	0	1	Endometritis	Hospital 1	China	Female
D412	36	3	0	2	Endometritis	Hospital 1	China	Female
D413	38	1	0	0	Endometritis	Hospital 1	China	Female
D414	32	2	0	1	Endometritis	Hospital 1	China	Female
D415	37	2	1	0	Endometritis	Hospital 1	China	Female
D416	37	1	0	1	Endometritis	Hospital 1	China	Female
D417	37	2	0	1	Endometritis	Hospital 1	China	Female
D418	37	1	0	0	Endometritis	Hospital 1	China	Female
D419	35	2	0	1	Endometritis	Hospital 1	China	Female
D420	36	2	0	1	Endometritis	Hospital 1	China	Female
D421	33	4	0	3	Endometritis	Hospital 1	China	Female

D422	37	2	0	0	Endometritis	Hospital 1	China	Female
D423	35	2	0	1	Endometritis	Hospital 1	China	Female
D424	37	2	0	1	Endometritis	Hospital 1	China	Female
D425	30	1	0	0	Endometritis	Hospital 1	China	Female
D426	37	1	0	1	Endometritis	Hospital 1	China	Female
D427	32	2	0	0	Endometritis	Hospital 1	China	Female
D428	36	1	0	0	Endometritis	Hospital 1	China	Female
D429	37	1	0	0	Endometritis	Hospital 1	China	Female
D430	36	1	0	0	Endometritis	Hospital 1	China	Female
D431	35	4	0	3	Endometritis	Hospital 1	China	Female
D432	37	2	0	1	Endometritis	Hospital 1	China	Female
D433	37	5	1	3	Endometritis	Hospital 1	China	Female
D434	36	1	0	0	Endometritis	Hospital 1	China	Female
D435	35	1	0	1	Endometritis	Hospital 1	China	Female
D436	37	2	0	2	Endometritis	Hospital 1	China	Female
D437	37	1	0	0	Endometritis	Hospital 1	China	Female
D438	34	1	0	1	Endometritis	Hospital 1	China	Female
D439	35	3	0	2	Endometritis	Hospital 1	China	Female
D440	37	2	0	1	Endometritis	Hospital 1	China	Female
D441	37	1	0	0	Endometritis	Hospital 1	China	Female
D442	33	3	1	1	Endometritis	Hospital 1	China	Female
D443	28	2	0	1	Endometritis	Hospital 1	China	Female
D444	37	1	0	0	Endometritis	Hospital 1	China	Female
D445	36	2	0	2	Endometritis	Hospital 1	China	Female
D446	37	2	0	2	Endometritis	Hospital 1	China	Female

D447	38	2	0	1	Endometritis	Hospital 1	China	Female
D448	37	2	0	0	Endometritis	Hospital 1	China	Female
D449	36	2	0	1	Endometritis	Hospital 1	China	Female
D450	37	2	0	2	Endometritis	Hospital 1	China	Female
D451	37	1	1	0	Endometritis	Hospital 1	China	Female
D452	37	1	0	0	Endometritis	Hospital 1	China	Female
D453	37	2	2	0	Endometritis	Hospital 1	China	Female
D454	37	4	2	2	Endometritis	Hospital 1	China	Female
D455	37	1	0	0	Endometritis	Hospital 1	China	Female
D456	36	2	0	2	Endometritis	Hospital 1	China	Female
D457	37	2	2	0	Endometritis	Hospital 1	China	Female
D458	34	1	1	0	Endometritis	Hospital 1	China	Female
D459	38	1	0	0	Endometritis	Hospital 1	China	Female
D460	37	2	2	0	Endometritis	Hospital 1	China	Female
D461	36	5	1	3	Endometritis	Hospital 1	China	Female
D462	37	3	1	1	Endometritis	Hospital 1	China	Female
D463	37	2	0	1	Endometritis	Hospital 1	China	Female
D464	35	3	1	1	Endometritis	Hospital 1	China	Female
D465	37	3	0	2	Endometritis	Hospital 1	China	Female
D466	37	1	1	0	Endometritis	Hospital 1	China	Female
D467	37	2	0	1	Endometritis	Hospital 1	China	Female
D468	37	2	1	0	Endometritis	Hospital 1	China	Female
D469	37	1	1	0	Endometritis	Hospital 1	China	Female
D470	37	2	0	1	Endometritis	Hospital 1	China	Female
D471	34	1	0	0	Endometritis	Hospital 1	China	Female

D472	31	2	0	1	Endometritis	Hospital 1	China	Female
D473	36	1	0	0	Endometritis	Hospital 1	China	Female
D474	38	2	0	2	Endometritis	Hospital 1	China	Female
D475	37	1	0	0	Endometritis	Hospital 1	China	Female
D476	36	1	0	1	Endometritis	Hospital 1	China	Female
D477	37	0	0	0	Endometritis	Hospital 1	China	Female
D478	35	3	0	3	Endometritis	Hospital 1	China	Female
D479	37	2	0	2	Endometritis	Hospital 1	China	Female
D480	37	1	0	1	Endometritis	Hospital 1	China	Female
D481	37	2	0	2	Endometritis	Hospital 1	China	Female
D482	37	2	0	1	Endometritis	Hospital 1	China	Female
D483	36	2	0	2	Endometritis	Hospital 1	China	Female
D484	37	1	0	0	Endometritis	Hospital 1	China	Female
D485	37	3	0	3	Endometritis	Hospital 1	China	Female
D486	38	2	0	1	Endometritis	Hospital 1	China	Female
D487	34	1	0	0	Endometritis	Hospital 1	China	Female
D488	37	3	0	1	Endometritis	Hospital 1	China	Female
D489	37	1	0	1	Endometritis	Hospital 1	China	Female
D490	36	1	0	0	Endometritis	Hospital 1	China	Female
D491	35	1	0	1	Endometritis	Hospital 1	China	Female
D492	37	2	1	1	Endometritis	Hospital 1	China	Female
D493	36	1	0	0	Endometritis	Hospital 1	China	Female
D494	28	2	0	1	Endometritis	Hospital 1	China	Female
D495	30	1	0	0	Endometritis	Hospital 1	China	Female
D496	37	2	0	1	Endometritis	Hospital 1	China	Female

D497	37	2	0	0	Endometritis	Hospital 1	China	Female
D498	35	1	0	0	Endometritis	Hospital 1	China	Female
D499	37	1	0	0	Endometritis	Hospital 1	China	Female
D500	37	4	1	3	Endometritis	Hospital 1	China	Female
H501	28	2	0	1	Health	Hospital 1	China	Female
H502	39	4	2	0	Health	Hospital 1	China	Female
H503	34	1	0	0	Health	Hospital 1	China	Female
H504	31	1	0	0	Health	Hospital 1	China	Female
H505	26	1	0	0	Health	Hospital 1	China	Female
H506	38	2	0	2	Health	Hospital 1	China	Female
H507	33	3	1	2	Health	Hospital 1	China	Female
H508	29	1	0	0	Health	Hospital 1	China	Female
H509	32	1	0	1	Health	Hospital 1	China	Female
H510	26	2	0	1	Health	Hospital 1	China	Female
H511	35	1	0	0	Health	Hospital 1	China	Female
H512	29	1	0	0	Health	Hospital 1	China	Female
H513	34	4	0	2	Health	Hospital 1	China	Female
H514	36	2	0	0	Health	Hospital 1	China	Female
H515	24	2	0	1	Health	Hospital 1	China	Female
H516	40	1	0	0	Health	Hospital 1	China	Female
H517	32	1	0	0	Health	Hospital 1	China	Female
H518	25	2	0	0	Health	Hospital 1	China	Female
H519	28	2	1	0	Health	Hospital 1	China	Female
H520	25	1	0	0	Health	Hospital 1	China	Female
H521	27	3	1	2	Health	Hospital 1	China	Female

H522	30	1	1	0	Health	Hospital 1	China	Female
H523	36	1	0	0	Health	Hospital 1	China	Female
H524	27	2	0	1	Health	Hospital 1	China	Female
H525	31	3	0	1	Health	Hospital 1	China	Female
H526	33	1	0	1	Health	Hospital 1	China	Female
H527	31	2	0	2	Health	Hospital 1	China	Female
H528	33	1	0	1	Health	Hospital 1	China	Female
H529	37	2	0	1	Health	Hospital 1	China	Female
H530	36	1	0	1	Health	Hospital 1	China	Female
H531	33	1	0	1	Health	Hospital 1	China	Female
H532	32	2	1	1	Health	Hospital 1	China	Female
H533	30	1	0	0	Health	Hospital 1	China	Female
H534	31	2	0	1	Health	Hospital 1	China	Female
H535	29	1	0	0	Health	Hospital 1	China	Female
H536	32	1	0	0	Health	Hospital 1	China	Female
H537	31	1	0	1	Health	Hospital 1	China	Female
H538	29	1	0	0	Health	Hospital 1	China	Female
H539	27	2	0	1	Health	Hospital 1	China	Female
H540	32	1	0	1	Health	Hospital 1	China	Female
H541	30	1	0	0	Health	Hospital 1	China	Female
H542	30	2	0	2	Health	Hospital 1	China	Female
H543	29	1	0	0	Health	Hospital 1	China	Female
H544	30	1	0	0	Health	Hospital 1	China	Female
H545	32	1	0	0	Health	Hospital 1	China	Female
H546	22	0	0	0	Health	Hospital 1	China	Female

H547	30	1	0	0	Health	Hospital 1	China	Female
H548	26	1	0	0	Health	Hospital 1	China	Female
H549	28	1	0	0	Health	Hospital 1	China	Female
H550	36	1	0	0	Health	Hospital 1	China	Female
H551	35	1	0	0	Health	Hospital 1	China	Female
H552	39	3	0	2	Health	Hospital 1	China	Female
H553	32	2	0	1	Health	Hospital 1	China	Female
H554	32	2	0	1	Health	Hospital 1	China	Female
H555	34	1	0	0	Health	Hospital 1	China	Female
H556	37	1	0	0	Health	Hospital 1	China	Female
H557	32	1	0	0	Health	Hospital 1	China	Female
H558	34	1	0	0	Health	Hospital 1	China	Female
H559	32	1	0	0	Health	Hospital 1	China	Female
H560	32	1	0	0	Health	Hospital 1	China	Female
H561	33	2	0	2	Health	Hospital 1	China	Female
H562	28	2	0	1	Health	Hospital 1	China	Female
H563	31	1	0	0	Health	Hospital 1	China	Female
H564	35	1	0	0	Health	Hospital 1	China	Female
H565	27	2	0	0	Health	Hospital 1	China	Female
H566	36	2	0	1	Health	Hospital 1	China	Female
H567	36	1	0	0	Health	Hospital 1	China	Female
H568	32	2	0	2	Health	Hospital 1	China	Female
H569	35	2	0	1	Health	Hospital 1	China	Female
H570	32	3	0	2	Health	Hospital 1	China	Female
H571	31	1	0	0	Health	Hospital 1	China	Female

H572	32	1	0	0	Health	Hospital 1	China	Female
H573	22	2	0	1	Health	Hospital 1	China	Female
H574	28	2	0	0	Health	Hospital 1	China	Female
H575	30	3	0	2	Health	Hospital 1	China	Female
H576	36	1	0	0	Health	Hospital 1	China	Female
H577	24	1	0	0	Health	Hospital 1	China	Female
H578	27	1	0	0	Health	Hospital 1	China	Female
H579	37	1	0	0	Health	Hospital 1	China	Female
H580	34	1	0	0	Health	Hospital 1	China	Female
H581	24	1	0	0	Health	Hospital 1	China	Female
H582	32	2	0	1	Health	Hospital 1	China	Female
H583	37	1	0	0	Health	Hospital 1	China	Female
H584	33	1	0	0	Health	Hospital 1	China	Female
H585	34	1	0	0	Health	Hospital 1	China	Female
H586	33	0	0	0	Health	Hospital 1	China	Female
H587	37	2	0	1	Health	Hospital 1	China	Female
H588	36	1	0	1	Health	Hospital 1	China	Female
H589	33	2	0	1	Health	Hospital 1	China	Female
H590	39	1	0	0	Health	Hospital 1	China	Female
H591	27	1	0	0	Health	Hospital 1	China	Female
H592	36	1	0	0	Health	Hospital 1	China	Female
H593	28	2	0	0	Health	Hospital 1	China	Female
H594	31	1	0	0	Health	Hospital 1	China	Female
H595	32	2	0	1	Health	Hospital 1	China	Female
H596	26	1	0	0	Health	Hospital 1	China	Female

H597	34	2	0	2	Health	Hospital 1	China	Female
H598	40	1	0	0	Health	Hospital 1	China	Female
H599	29	2	0	1	Health	Hospital 1	China	Female
H600	37	2	0	2	Health	Hospital 1	China	Female
H601	29	3	1	1	Health	Hospital 1	China	Female
H602	39	1	0	0	Health	Hospital 1	China	Female
H603	36	2	0	0	Health	Hospital 1	China	Female
H604	29	3	0	2	Health	Hospital 1	China	Female
H605	35	3	0	2	Health	Hospital 1	China	Female
H606	28	2	0	1	Health	Hospital 1	China	Female
H607	27	1	0	0	Health	Hospital 1	China	Female
H608	35	1	0	0	Health	Hospital 1	China	Female
H609	27	2	0	1	Health	Hospital 1	China	Female
H610	31	2	0	0	Health	Hospital 1	China	Female
H611	30	1	0	0	Health	Hospital 1	China	Female
H612	29	2	0	1	Health	Hospital 1	China	Female
H613	22	1	0	1	Health	Hospital 1	China	Female
H614	31	1	0	0	Health	Hospital 1	China	Female
H615	32	2	1	0	Health	Hospital 1	China	Female
H616	36	3	0	2	Health	Hospital 1	China	Female
H617	36	1	0	0	Health	Hospital 1	China	Female
H618	32	1	0	0	Health	Hospital 1	China	Female
H619	30	1	0	0	Health	Hospital 1	China	Female
H620	32	1	0	0	Health	Hospital 1	China	Female
H621	27	1	0	0	Health	Hospital 1	China	Female

H622	25	3	0	2	Health	Hospital 1	China	Female
H623	33	1	0	1	Health	Hospital 1	China	Female
H624	24	2	0	1	Health	Hospital 1	China	Female
H625	23	1	0	0	Health	Hospital 1	China	Female
H626	21	2	0	1	Health	Hospital 1	China	Female
H627	32	3	0	2	Health	Hospital 1	China	Female
H628	47	2	0	0	Health	Hospital 1	China	Female
H629	39	1	0	0	Health	Hospital 1	China	Female
H630	28	3	0	1	Health	Hospital 1	China	Female
H631	32	2	0	1	Health	Hospital 1	China	Female
H632	31	3	0	2	Health	Hospital 1	China	Female
H633	30	2	0	0	Health	Hospital 1	China	Female
H634	27	3	0	2	Health	Hospital 1	China	Female
H635	26	1	0	0	Health	Hospital 1	China	Female
H636	26	2	0	0	Health	Hospital 1	China	Female
H637	20	2	0	1	Health	Hospital 1	China	Female
H638	23	2	0	1	Health	Hospital 1	China	Female
H639	38	1	0	0	Health	Hospital 1	China	Female
H640	36	3	0	0	Health	Hospital 1	China	Female
H641	35	2	0	0	Health	Hospital 1	China	Female
H642	31	1	0	0	Health	Hospital 1	China	Female
H643	34	1	0	0	Health	Hospital 1	China	Female
H644	24	1	0	0	Health	Hospital 1	China	Female
H645	27	1	0	0	Health	Hospital 1	China	Female
H646	41	1	0	0	Health	Hospital 1	China	Female

H647	34	3	0	3	Health	Hospital 1	China	Female
H648	24	3	0	2	Health	Hospital 1	China	Female
H649	32	1	0	1	Health	Hospital 1	China	Female
H650	34	1	0	0	Health	Hospital 1	China	Female
H651	31	1	0	0	Health	Hospital 1	China	Female
H652	26	1	0	0	Health	Hospital 1	China	Female
H653	38	1	0	1	Health	Hospital 1	China	Female
H654	33	2	0	2	Health	Hospital 1	China	Female
H655	29	1	0	0	Health	Hospital 1	China	Female
H656	38	1	0	0	Health	Hospital 1	China	Female
H657	26	3	0	1	Health	Hospital 1	China	Female
H658	27	3	0	2	Health	Hospital 1	China	Female
H659	28	2	0	0	Health	Hospital 1	China	Female
H660	26	3	0	2	Health	Hospital 1	China	Female
H661	31	2	0	2	Health	Hospital 1	China	Female
H662	34	4	0	3	Health	Hospital 1	China	Female
H663	29	2	0	1	Health	Hospital 1	China	Female
H664	40	2	0	0	Health	Hospital 1	China	Female
H665	32	1	0	0	Health	Hospital 1	China	Female
H666	28	1	0	1	Health	Hospital 1	China	Female
H667	36	3	0	0	Health	Hospital 1	China	Female
H668	36	1	0	0	Health	Hospital 1	China	Female
H669	26	3	1	0	Health	Hospital 1	China	Female
H670	33	3	0	1	Health	Hospital 1	China	Female
H671	36	4	0	3	Health	Hospital 1	China	Female

H672	27	1	0	0	Health	Hospital 1	China	Female
H673	36	1	0	0	Health	Hospital 1	China	Female
H674	29	2	0	0	Health	Hospital 1	China	Female
H675	45	1	0	0	Health	Hospital 1	China	Female
H676	28	2	0	0	Health	Hospital 1	China	Female
H677	31	2	0	1	Health	Hospital 1	China	Female
H678	32	1	0	0	Health	Hospital 1	China	Female
H679	28	1	0	0	Health	Hospital 1	China	Female
H680	25	1	1	0	Health	Hospital 1	China	Female
H681	35	1	0	0	Health	Hospital 1	China	Female
H682	33	2	0	0	Health	Hospital 1	China	Female
H683	28	1	0	0	Health	Hospital 1	China	Female
H684	29	1	0	0	Health	Hospital 1	China	Female
H685	27	1	0	0	Health	Hospital 1	China	Female
H686	29	2	0	1	Health	Hospital 1	China	Female
H687	30	2	0	1	Health	Hospital 1	China	Female
H688	30	1	0	0	Health	Hospital 1	China	Female
H689	33	2	0	0	Health	Hospital 1	China	Female
H690	37	2	0	0	Health	Hospital 1	China	Female
H691	36	2	0	0	Health	Hospital 1	China	Female
H692	33	2	1	0	Health	Hospital 1	China	Female
H693	32	1	0	0	Health	Hospital 1	China	Female
H694	30	1	0	0	Health	Hospital 1	China	Female
H695	28	2	1	2	Health	Hospital 1	China	Female
H696	30	1	0	0	Health	Hospital 1	China	Female

H697	39	2	0	2	Health	Hospital 1	China	Female
H698	34	2	0	0	Health	Hospital 1	China	Female
H699	32	3	0	2	Health	Hospital 1	China	Female
H700	31	1	0	0	Health	Hospital 1	China	Female
H701	32	1	0	0	Health	Hospital 1	China	Female
H702	26	2	0	2	Health	Hospital 1	China	Female
H703	34	3	0	1	Health	Hospital 1	China	Female
H704	28	1	0	1	Health	Hospital 1	China	Female
H705	36	1	0	0	Health	Hospital 1	China	Female
H706	29	3	0	2	Health	Hospital 1	China	Female
H707	29	1	0	0	Health	Hospital 1	China	Female
H708	32	2	0	1	Health	Hospital 1	China	Female
H709	32	4	0	2	Health	Hospital 1	China	Female
H710	25	1	0	0	Health	Hospital 1	China	Female
H711	35	3	0	1	Health	Hospital 1	China	Female
H712	34	1	0	0	Health	Hospital 1	China	Female
H713	40	1	0	0	Health	Hospital 1	China	Female
H714	21	4	0	0	Health	Hospital 1	China	Female
H715	28	1	0	0	Health	Hospital 1	China	Female
H716	30	2	0	0	Health	Hospital 1	China	Female
H717	22	4	0	0	Health	Hospital 1	China	Female
H718	30	2	0	0	Health	Hospital 1	China	Female
H719	33	4	0	1	Health	Hospital 1	China	Female
H720	35	1	0	0	Health	Hospital 1	China	Female
H721	28	1	0	0	Health	Hospital 1	China	Female

H722	38	2	0	0	Health	Hospital 1	China	Female
H723	26	1	0	0	Health	Hospital 1	China	Female
H724	34	1	0	0	Health	Hospital 1	China	Female
H725	28	1	0	0	Health	Hospital 1	China	Female
H726	36	1	0	0	Health	Hospital 1	China	Female
H727	34	3	0	1	Health	Hospital 1	China	Female
H728	38	3	0	1	Health	Hospital 1	China	Female
H729	38	1	0	0	Health	Hospital 1	China	Female
H730	34	2	0	0	Health	Hospital 1	China	Female
H731	31	1	0	0	Health	Hospital 1	China	Female
H732	29	4	0	0	Health	Hospital 1	China	Female
H733	32	3	0	2	Health	Hospital 1	China	Female
H734	33	1	0	0	Health	Hospital 1	China	Female
H735	32	2	0	2	Health	Hospital 1	China	Female
H736	34	1	0	0	Health	Hospital 1	China	Female
H737	37	1	0	0	Health	Hospital 1	China	Female
H738	25	1	0	0	Health	Hospital 1	China	Female
H739	29	2	0	0	Health	Hospital 1	China	Female
H740	36	2	0	1	Health	Hospital 1	China	Female
H741	34	1	0	0	Health	Hospital 1	China	Female
H742	37	1	0	0	Health	Hospital 1	China	Female
H743	28	1	0	0	Health	Hospital 1	China	Female
H744	26	1	0	0	Health	Hospital 1	China	Female
H745	27	1	0	0	Health	Hospital 1	China	Female
H746	27	2	0	1	Health	Hospital 1	China	Female

H747	23	1	0	1	Health	Hospital 1	China	Female
H748	33	1	0	1	Health	Hospital 1	China	Female
H749	28	1	0	1	Health	Hospital 1	China	Female
H750	29	1	0	0	Health	Hospital 1	China	Female
H751	30	1	0	0	Health	Hospital 1	China	Female
H752	31	1	0	0	Health	Hospital 1	China	Female
H753	29	2	0	1	Health	Hospital 1	China	Female
H754	35	1	1	0	Health	Hospital 1	China	Female
H755	33	1	0	0	Health	Hospital 1	China	Female
H756	29	1	0	0	Health	Hospital 1	China	Female
H757	39	1	0	0	Health	Hospital 1	China	Female
H758	38	2	0	0	Health	Hospital 1	China	Female
H759	37	3	0	3	Health	Hospital 1	China	Female
H760	28	2	0	2	Health	Hospital 1	China	Female
H761	29	4	1	3	Health	Hospital 1	China	Female
H762	32	2	0	2	Health	Hospital 1	China	Female
H763	28	1	0	0	Health	Hospital 1	China	Female
H764	35	1	0	0	Health	Hospital 1	China	Female
H765	34	3	0	0	Health	Hospital 1	China	Female
H766	24	1	0	0	Health	Hospital 1	China	Female
H767	30	1	0	0	Health	Hospital 1	China	Female
H768	34	2	0	2	Health	Hospital 1	China	Female
H769	27	2	0	1	Health	Hospital 1	China	Female
H770	37	1	0	1	Health	Hospital 1	China	Female
H771	34	2	0	0	Health	Hospital 1	China	Female

H772	30	1	0	1	Health	Hospital 1	China	Female
H773	33	3	0	2	Health	Hospital 1	China	Female
H774	27	2	0	0	Health	Hospital 1	China	Female
H775	37	4	1	3	Health	Hospital 1	China	Female
H776	33	1	0	0	Health	Hospital 1	China	Female
H777	37	1	0	0	Health	Hospital 1	China	Female
H778	32	1	0	0	Health	Hospital 1	China	Female
H779	29	2	0	0	Health	Hospital 1	China	Female
H780	34	1	0	0	Health	Hospital 1	China	Female
H781	37	4	0	2	Health	Hospital 1	China	Female
H782	36	3	0	0	Health	Hospital 1	China	Female
H783	37	1	0	0	Health	Hospital 1	China	Female
H784	24	2	0	0	Health	Hospital 1	China	Female
H785	26	1	0	0	Health	Hospital 1	China	Female
H786	29	1	0	0	Health	Hospital 1	China	Female
H787	30	2	0	1	Health	Hospital 1	China	Female
H788	30	2	0	0	Health	Hospital 1	China	Female
H789	33	2	0	0	Health	Hospital 1	China	Female
H790	29	1	0	0	Health	Hospital 1	China	Female
H791	33	2	0	0	Health	Hospital 1	China	Female
H792	36	1	0	0	Health	Hospital 1	China	Female
H793	25	2	0	0	Health	Hospital 1	China	Female
H794	33	2	0	0	Health	Hospital 1	China	Female
H795	35	2	0	0	Health	Hospital 1	China	Female
H796	29	3	1	0	Health	Hospital 1	China	Female

H797	31	3	0	0	Health	Hospital 1	China	Female
H798	36	2	1	0	Health	Hospital 1	China	Female
H799	35	2	1	0	Health	Hospital 1	China	Female
H800	26	3	0	0	Health	Hospital 1	China	Female
H801	24	2	0	0	Health	Hospital 1	China	Female
H802	31	1	0	0	Health	Hospital 1	China	Female
H803	29	2	1	0	Health	Hospital 1	China	Female
H804	31	1	0	0	Health	Hospital 1	China	Female
H805	30	3	0	2	Health	Hospital 1	China	Female
H806	29	1	0	0	Health	Hospital 1	China	Female
H807	28	3	1	0	Health	Hospital 1	China	Female
H808	32	1	0	0	Health	Hospital 1	China	Female
H809	26	1	1	0	Health	Hospital 1	China	Female
H810	27	1	1	0	Health	Hospital 1	China	Female
H811	41	2	0	1	Health	Hospital 1	China	Female
H812	29	2	0	0	Health	Hospital 1	China	Female
H813	25	1	0	1	Health	Hospital 1	China	Female
H814	30	2	0	0	Health	Hospital 1	China	Female
H815	25	5	0	1	Health	Hospital 1	China	Female
H816	30	1	1	0	Health	Hospital 1	China	Female
H817	25	2	0	0	Health	Hospital 1	China	Female
H818	32	2	0	0	Health	Hospital 1	China	Female
H819	32	3	1	0	Health	Hospital 1	China	Female
H820	32	1	0	0	Health	Hospital 1	China	Female
H821	34	1	0	0	Health	Hospital 1	China	Female

H822	26	2	0	1	Health	Hospital 1	China	Female
H823	30	1	0	0	Health	Hospital 1	China	Female
H824	28	3	1	0	Health	Hospital 1	China	Female
H825	33	2	0	0	Health	Hospital 1	China	Female
H826	29	1	0	0	Health	Hospital 1	China	Female
H827	37	3	0	0	Health	Hospital 1	China	Female
H828	32	2	0	0	Health	Hospital 1	China	Female
H829	32	4	0	0	Health	Hospital 1	China	Female
H830	31	3	0	1	Health	Hospital 1	China	Female
H831	39	2	0	0	Health	Hospital 1	China	Female
H832	31	1	0	0	Health	Hospital 1	China	Female
H833	37	2	1	0	Health	Hospital 1	China	Female
H834	36	1	0	0	Health	Hospital 1	China	Female
H835	34	2	0	0	Health	Hospital 1	China	Female
H836	37	1	1	0	Health	Hospital 1	China	Female
H837	33	2	0	0	Health	Hospital 1	China	Female
H838	33	1	0	0	Health	Hospital 1	China	Female
H839	33	1	0	0	Health	Hospital 1	China	Female
H840	29	3	0	0	Health	Hospital 1	China	Female
H841	33	4	1	0	Health	Hospital 1	China	Female
H842	35	1	0	0	Health	Hospital 1	China	Female
H843	32	1	0	0	Health	Hospital 1	China	Female
H844	30	1	0	0	Health	Hospital 1	China	Female
H845	32	1	0	0	Health	Hospital 1	China	Female
H846	32	2	0	0	Health	Hospital 1	China	Female

H847	38	1	0	0	Health	Hospital 1	China	Female
H848	43	3	2	0	Health	Hospital 1	China	Female
H849	26	3	0	0	Health	Hospital 1	China	Female
H850	33	1	0	0	Health	Hospital 1	China	Female
H851	37	5	0	0	Health	Hospital 1	China	Female
H852	34	5	0	0	Health	Hospital 1	China	Female
H853	27	1	0	0	Health	Hospital 1	China	Female
H854	25	1	0	0	Health	Hospital 1	China	Female
H855	23	1	0	0	Health	Hospital 1	China	Female
H856	29	2	0	0	Health	Hospital 1	China	Female
H857	31	2	0	1	Health	Hospital 1	China	Female
H858	28	2	0	1	Health	Hospital 1	China	Female
H859	30	4	0	1	Health	Hospital 1	China	Female
H860	34	2	0	1	Health	Hospital 1	China	Female
H861	28	2	0	0	Health	Hospital 1	China	Female
H862	35	1	0	0	Health	Hospital 1	China	Female
H863	24	0	0	0	Health	Hospital 1	China	Female
H864	23	2	0	1	Health	Hospital 1	China	Female
H865	29	1	0	0	Health	Hospital 1	China	Female
H866	25	2	0	1	Health	Hospital 1	China	Female
H867	26	0	0	0	Health	Hospital 1	China	Female
H868	36	5	0	4	Health	Hospital 1	China	Female
H869	27	1	1	0	Health	Hospital 1	China	Female
H870	33	4	0	0	Health	Hospital 1	China	Female
H871	28	2	1	0	Health	Hospital 1	China	Female

H872	37	1	0	0	Health	Hospital 1	China	Female
H873	28	1	1	0	Health	Hospital 1	China	Female
H874	43	3	0	2	Health	Hospital 1	China	Female
H875	34	4	1	1	Health	Hospital 1	China	Female
H876	29	1	0	0	Health	Hospital 1	China	Female
H877	41	2	0	0	Health	Hospital 1	China	Female
H878	26	1	0	0	Health	Hospital 1	China	Female
H879	32	4	0	0	Health	Hospital 1	China	Female
H880	28	1	1	0	Health	Hospital 1	China	Female
H881	32	2	0	0	Health	Hospital 1	China	Female
H882	34	2	0	0	Health	Hospital 1	China	Female
H883	33	4	0	3	Health	Hospital 1	China	Female
H884	30	2	0	1	Health	Hospital 1	China	Female
H885	33	1	1	0	Health	Hospital 1	China	Female
H886	18	1	0	0	Health	Hospital 1	China	Female
H887	29	1	0	0	Health	Hospital 1	China	Female
H888	30	3	1	2	Health	Hospital 1	China	Female
H889	37	1	0	0	Health	Hospital 1	China	Female
H890	35	3	0	1	Health	Hospital 1	China	Female
H891	32	2	0	0	Health	Hospital 1	China	Female
H892	37	2	0	2	Health	Hospital 1	China	Female
H893	34	1	0	0	Health	Hospital 1	China	Female
H894	31	1	0	0	Health	Hospital 1	China	Female
H895	32	3	0	2	Health	Hospital 1	China	Female
H896	37	1	1	0	Health	Hospital 1	China	Female

H897	27	3	0	0	Health	Hospital 1	China	Female
H898	35	1	0	0	Health	Hospital 1	China	Female
H899	31	1	0	0	Health	Hospital 1	China	Female
H900	28	1	0	0	Health	Hospital 1	China	Female
H901	34	4	1	3	Health	Hospital 1	China	Female
H902	26	1	0	0	Health	Hospital 1	China	Female
H903	29	4	0	0	Health	Hospital 1	China	Female
H904	34	1	0	0	Health	Hospital 1	China	Female
H905	29	1	0	0	Health	Hospital 1	China	Female
H906	28	1	0	0	Health	Hospital 1	China	Female
H907	31	2	0	2	Health	Hospital 1	China	Female
H908	35	1	0	0	Health	Hospital 1	China	Female
H909	30	1	0	1	Health	Hospital 1	China	Female
H910	32	1	0	0	Health	Hospital 1	China	Female
H911	36	2	0	1	Health	Hospital 1	China	Female
H912	25	2	1	0	Health	Hospital 1	China	Female
H913	28	4	0	3	Health	Hospital 1	China	Female
H914	38	1	0	0	Health	Hospital 1	China	Female
H915	30	1	0	0	Health	Hospital 1	China	Female
H916	36	3	0	2	Health	Hospital 1	China	Female
H917	29	2	0	1	Health	Hospital 1	China	Female
H918	25	1	0	0	Health	Hospital 1	China	Female
H919	33	2	0	1	Health	Hospital 1	China	Female
H920	34	2	0	1	Health	Hospital 1	China	Female
H921	37	1	0	0	Health	Hospital 1	China	Female

H922	29	2	1	1	Health	Hospital 1	China	Female
H923	33	2	0	0	Health	Hospital 1	China	Female
H924	27	3	0	2	Health	Hospital 1	China	Female
H925	32	3	0	2	Health	Hospital 1	China	Female
H926	32	6	0	3	Health	Hospital 1	China	Female
H927	28	3	0	2	Health	Hospital 1	China	Female
H928	31	1	0	0	Health	Hospital 1	China	Female
H929	32	3	0	2	Health	Hospital 1	China	Female
H930	34	1	0	0	Health	Hospital 1	China	Female
H931	29	1	0	0	Health	Hospital 1	China	Female
H932	28	2	0	1	Health	Hospital 1	China	Female
H933	30	1	0	0	Health	Hospital 1	China	Female
H934	29	5	0	0	Health	Hospital 1	China	Female
H935	35	1	0	0	Health	Hospital 1	China	Female
H936	28	3	0	2	Health	Hospital 1	China	Female
H937	30	1	1	0	Health	Hospital 1	China	Female
H938	35	2	0	1	Health	Hospital 1	China	Female
H939	27	2	0	1	Health	Hospital 1	China	Female
H940	35	1	0	0	Health	Hospital 1	China	Female
H941	32	4	1	2	Health	Hospital 1	China	Female
H942	32	3	0	1	Health	Hospital 1	China	Female
H943	29	1	0	0	Health	Hospital 1	China	Female
H944	24	2	0	0	Health	Hospital 1	China	Female
H945	31	3	0	0	Health	Hospital 1	China	Female
H946	35	1	0	0	Health	Hospital 1	China	Female

H947	30	1	0	0	Health	Hospital 1	China	Female
H948	27	3	0	2	Health	Hospital 1	China	Female
H949	39	1	0	0	Health	Hospital 1	China	Female
H950	33	1	0	0	Health	Hospital 1	China	Female
H951	32	3	0	1	Health	Hospital 1	China	Female
H952	35	2	0	0	Health	Hospital 1	China	Female
H953	33	2	0	0	Health	Hospital 1	China	Female
H954	28	2	0	0	Health	Hospital 1	China	Female
H955	32	2	0	0	Health	Hospital 1	China	Female
H956	29	1	0	0	Health	Hospital 1	China	Female
H957	31	3	0	2	Health	Hospital 1	China	Female
H958	30	1	0	0	Health	Hospital 1	China	Female
H959	26	1	0	0	Health	Hospital 1	China	Female
H960	25	1	0	0	Health	Hospital 1	China	Female
H961	31	1	0	0	Health	Hospital 1	China	Female
H962	24	4	0	1	Health	Hospital 1	China	Female
H963	36	1	0	1	Health	Hospital 1	China	Female
H964	28	2	0	1	Health	Hospital 1	China	Female
H965	36	3	0	2	Health	Hospital 1	China	Female
H966	34	4	0	3	Health	Hospital 1	China	Female
H967	32	1	0	0	Health	Hospital 1	China	Female
H968	29	2	0	1	Health	Hospital 1	China	Female
H969	37	2	0	1	Health	Hospital 1	China	Female
H970	38	1	0	0	Health	Hospital 1	China	Female
H971	23	3	1	1	Health	Hospital 1	China	Female

H972	39	1	0	0	Health	Hospital 1	China	Female
H973	35	1	0	0	Health	Hospital 1	China	Female
H974	31	1	0	0	Health	Hospital 1	China	Female
H975	31	3	0	2	Health	Hospital 1	China	Female
H976	40	5	0	4	Health	Hospital 1	China	Female
H977	28	2	0	1	Health	Hospital 1	China	Female
H978	32	4	0	2	Health	Hospital 1	China	Female
H979	38	1	0	1	Health	Hospital 1	China	Female
H980	28	2	0	1	Health	Hospital 1	China	Female
H981	29	1	0	0	Health	Hospital 1	China	Female
H982	29	1	1	0	Health	Hospital 1	China	Female
H983	33	2	0	1	Health	Hospital 1	China	Female
H984	28	1	0	1	Health	Hospital 1	China	Female
H985	41	2	0	1	Health	Hospital 1	China	Female
H986	38	2	0	1	Health	Hospital 1	China	Female
H987	29	1	0	0	Health	Hospital 1	China	Female
H988	29	1	0	0	Health	Hospital 1	China	Female
H989	28	2	0	1	Health	Hospital 1	China	Female
H990	38	2	0	0	Health	Hospital 1	China	Female
H991	31	1	0	0	Health	Hospital 1	China	Female
H992	30	2	1	0	Health	Hospital 1	China	Female
H993	28	1	0	0	Health	Hospital 1	China	Female
H994	33	2	0	2	Health	Hospital 1	China	Female
H995	40	1	0	0	Health	Hospital 1	China	Female
H996	32	3	0	2	Health	Hospital 1	China	Female

H997	30	1	0	1	Health	Hospital 1	China	Female
H998	35	1	0	0	Health	Hospital 1	China	Female
H999	20	2	0	2	Health	Hospital 1	China	Female
H1000	27	1	0	0	Health	Hospital 1	China	Female
D1001	32	-	-	-	Endometritis	Hospital 2	China	Female
D1002	38	-	-	-	Endometritis	Hospital 2	China	Female
D1003	41	-	-	-	Endometritis	Hospital 2	China	Female
D1004	53	-	-	-	Endometritis	Hospital 2	China	Female
D1005	55	-	-	-	Endometritis	Hospital 2	China	Female
D1006	30	-	-	-	Endometritis	Hospital 2	China	Female
D1007	58	-	-	-	Endometritis	Hospital 2	China	Female
D1008	31	-	-	-	Endometritis	Hospital 2	China	Female
D1009	39	-	-	-	Endometritis	Hospital 2	China	Female
D1010	35	-	-	-	Endometritis	Hospital 2	China	Female
D1011	39	-	-	-	Endometritis	Hospital 2	China	Female
D1012	37	-	-	-	Endometritis	Hospital 2	China	Female
D1013	40	-	-	-	Endometritis	Hospital 2	China	Female
D1014	31	-	-	-	Endometritis	Hospital 2	China	Female
D1015	51	-	-	-	Endometritis	Hospital 2	China	Female
D1016	48	-	-	-	Endometritis	Hospital 2	China	Female
D1017	30	-	-	-	Endometritis	Hospital 2	China	Female
D1018	32	-	-	-	Endometritis	Hospital 2	China	Female
D1019	38	-	-	-	Endometritis	Hospital 2	China	Female
D1020	38	-	-	-	Endometritis	Hospital 2	China	Female
D1021	41	-	-	-	Endometritis	Hospital 2	China	Female

D1022	32	-	-	-	Endometritis	Hospital 2	China	Female
D1023	34	-	-	-	Endometritis	Hospital 2	China	Female
D1024	42	-	-	-	Endometritis	Hospital 2	China	Female
D1025	71	-	-	-	Endometritis	Hospital 2	China	Female
D1026	63	-	-	-	Endometritis	Hospital 2	China	Female
D1027	65	-	-	-	Endometritis	Hospital 2	China	Female
D1028	40	-	-	-	Endometritis	Hospital 2	China	Female
D1029	36	-	-	-	Endometritis	Hospital 2	China	Female
D1030	43	-	-	-	Endometritis	Hospital 2	China	Female
D1031	35	-	-	-	Endometritis	Hospital 2	China	Female
D1032	31	-	-	-	Endometritis	Hospital 2	China	Female
D1033	30	-	-	-	Endometritis	Hospital 2	China	Female
D1034	42	-	-	-	Endometritis	Hospital 2	China	Female
D1035	32	-	-	-	Endometritis	Hospital 2	China	Female
D1036	42	-	-	-	Endometritis	Hospital 2	China	Female
D1037	53	-	-	-	Endometritis	Hospital 2	China	Female
D1038	40	-	-	-	Endometritis	Hospital 2	China	Female
D1039	59	-	-	-	Endometritis	Hospital 2	China	Female
D1040	39	-	-	-	Endometritis	Hospital 2	China	Female
D1041	28	-	-	-	Endometritis	Hospital 2	China	Female
D1042	43	-	-	-	Endometritis	Hospital 2	China	Female
D1043	60	-	-	-	Endometritis	Hospital 2	China	Female
D1044	42	-	-	-	Endometritis	Hospital 2	China	Female
D1045	54	-	-	-	Endometritis	Hospital 2	China	Female
D1046	37	-	-	-	Endometritis	Hospital 2	China	Female

D1047	50	-	-	-	Endometritis	Hospital 2	China	Female
D1048	33	-	-	-	Endometritis	Hospital 2	China	Female
D1049	59	-	-	-	Endometritis	Hospital 2	China	Female
D1050	56	-	-	-	Endometritis	Hospital 2	China	Female
D1051	38	-	-	-	Endometritis	Hospital 2	China	Female
D1052	33	-	-	-	Endometritis	Hospital 2	China	Female
D1053	47	-	-	-	Endometritis	Hospital 2	China	Female
D1054	32	-	-	-	Endometritis	Hospital 2	China	Female
D1055	52	-	-	-	Endometritis	Hospital 2	China	Female
D1056	49	-	-	-	Endometritis	Hospital 2	China	Female
D1057	43	-	-	-	Endometritis	Hospital 2	China	Female
D1058	34	-	-	-	Endometritis	Hospital 2	China	Female
D1059	36	-	-	-	Endometritis	Hospital 2	China	Female
D1060	32	-	-	-	Endometritis	Hospital 2	China	Female
D1061	58	-	-	-	Endometritis	Hospital 2	China	Female
D1062	32	-	-	-	Endometritis	Hospital 2	China	Female
D1063	33	-	-	-	Endometritis	Hospital 2	China	Female
D1064	40	-	-	-	Endometritis	Hospital 2	China	Female
D1065	35	-	-	-	Endometritis	Hospital 2	China	Female
D1066	25	-	-	-	Endometritis	Hospital 2	China	Female
D1067	48	-	-	-	Endometritis	Hospital 2	China	Female
D1068	39	-	-	-	Endometritis	Hospital 2	China	Female
D1069	54	-	-	-	Endometritis	Hospital 2	China	Female
D1070	60	-	-	-	Endometritis	Hospital 2	China	Female
D1071	41	-	-	-	Endometritis	Hospital 2	China	Female

D1072	43	-	-	-	Endometritis	Hospital 2	China	Female
D1073	48	-	-	-	Endometritis	Hospital 2	China	Female
D1074	37	-	-	-	Endometritis	Hospital 2	China	Female
D1075	52	-	-	-	Endometritis	Hospital 2	China	Female
D1076	32	-	-	-	Endometritis	Hospital 2	China	Female
D1077	53	-	-	-	Endometritis	Hospital 2	China	Female
D1078	31	-	-	-	Endometritis	Hospital 2	China	Female
D1079	47	-	-	-	Endometritis	Hospital 2	China	Female
D1080	41	-	-	-	Endometritis	Hospital 2	China	Female
D1081	49	-	-	-	Endometritis	Hospital 2	China	Female
D1082	52	-	-	-	Endometritis	Hospital 2	China	Female
D1083	45	-	-	-	Endometritis	Hospital 2	China	Female
D1084	40	-	-	-	Endometritis	Hospital 2	China	Female
D1085	34	-	-	-	Endometritis	Hospital 2	China	Female
D1086	31	-	-	-	Endometritis	Hospital 2	China	Female
D1087	42	-	-	-	Endometritis	Hospital 2	China	Female
D1088	43	-	-	-	Endometritis	Hospital 2	China	Female
D1089	31	-	-	-	Endometritis	Hospital 2	China	Female
D1090	55	-	-	-	Endometritis	Hospital 2	China	Female
D1091	37	-	-	-	Endometritis	Hospital 2	China	Female
D1092	30	-	-	-	Endometritis	Hospital 2	China	Female
D1093	26	-	-	-	Endometritis	Hospital 2	China	Female
D1094	34	-	-	-	Endometritis	Hospital 2	China	Female
D1095	50	-	-	-	Endometritis	Hospital 2	China	Female
D1096	34	-	-	-	Endometritis	Hospital 2	China	Female

D1097	65	-	-	-	Endometritis	Hospital 2	China	Female
D1098	37	-	-	-	Endometritis	Hospital 2	China	Female
D1099	42	-	-	-	Endometritis	Hospital 2	China	Female
D1100	51	-	-	-	Endometritis	Hospital 2	China	Female
D1101	34	-	-	-	Endometritis	Hospital 2	China	Female
D1102	45	-	-	-	Endometritis	Hospital 2	China	Female
D1103	39	-	-	-	Endometritis	Hospital 2	China	Female
D1104	43	-	-	-	Endometritis	Hospital 2	China	Female
D1105	32	-	-	-	Endometritis	Hospital 2	China	Female
D1106	41	-	-	-	Endometritis	Hospital 2	China	Female
D1107	38	-	-	-	Endometritis	Hospital 2	China	Female
D1108	37	-	-	-	Endometritis	Hospital 2	China	Female
D1109	34	-	-	-	Endometritis	Hospital 2	China	Female
D1110	38	-	-	-	Endometritis	Hospital 2	China	Female
D1111	31	-	-	-	Endometritis	Hospital 2	China	Female
D1112	40	-	-	-	Endometritis	Hospital 2	China	Female
D1113	31	-	-	-	Endometritis	Hospital 2	China	Female
D1114	54	-	-	-	Endometritis	Hospital 2	China	Female
D1115	40	-	-	-	Endometritis	Hospital 2	China	Female
D1116	39	-	-	-	Endometritis	Hospital 2	China	Female
D1117	33	-	-	-	Endometritis	Hospital 2	China	Female
D1118	33	-	-	-	Endometritis	Hospital 2	China	Female
D1119	63	-	-	-	Endometritis	Hospital 2	China	Female
D1120	33	-	-	-	Endometritis	Hospital 2	China	Female
D1121	48	-	-	-	Endometritis	Hospital 2	China	Female

D1122	34	-	-	-	Endometritis	Hospital 2	China	Female
D1123	66	-	-	-	Endometritis	Hospital 2	China	Female
D1124	46	-	-	-	Endometritis	Hospital 2	China	Female
D1125	36	-	-	-	Endometritis	Hospital 2	China	Female
D1126	50	-	-	-	Endometritis	Hospital 2	China	Female
D1127	44	-	-	-	Endometritis	Hospital 2	China	Female
D1128	38	-	-	-	Endometritis	Hospital 2	China	Female
D1129	36	-	-	-	Endometritis	Hospital 2	China	Female
D1130	39	-	-	-	Endometritis	Hospital 2	China	Female
D1131	49	-	-	-	Endometritis	Hospital 2	China	Female
D1132	35	-	-	-	Endometritis	Hospital 2	China	Female
D1133	32	-	-	-	Endometritis	Hospital 2	China	Female
D1134	39	-	-	-	Endometritis	Hospital 2	China	Female
D1135	51	-	-	-	Endometritis	Hospital 2	China	Female
D1136	41	-	-	-	Endometritis	Hospital 2	China	Female
D1137	56	-	-	-	Endometritis	Hospital 2	China	Female
D1138	35	-	-	-	Endometritis	Hospital 2	China	Female
D1139	50	-	-	-	Endometritis	Hospital 2	China	Female
D1140	43	-	-	-	Endometritis	Hospital 2	China	Female
D1141	44	-	-	-	Endometritis	Hospital 2	China	Female
D1142	30	-	-	-	Endometritis	Hospital 2	China	Female
D1143	60	-	-	-	Endometritis	Hospital 2	China	Female
D1144	43	-	-	-	Endometritis	Hospital 2	China	Female
D1145	30	-	-	-	Endometritis	Hospital 2	China	Female
D1146	43	-	-	-	Endometritis	Hospital 2	China	Female

D1147	33	-	-	-	Endometritis	Hospital 2	China	Female
D1148	36	-	-	-	Endometritis	Hospital 2	China	Female
D1149	67	-	-	-	Endometritis	Hospital 2	China	Female
D1150	47	-	-	-	Endometritis	Hospital 2	China	Female
D1151	40	-	-	-	Endometritis	Hospital 2	China	Female
D1152	43	-	-	-	Endometritis	Hospital 2	China	Female
D1153	38	-	-	-	Endometritis	Hospital 2	China	Female
D1154	38	-	-	-	Endometritis	Hospital 2	China	Female
D1155	52	-	-	-	Endometritis	Hospital 2	China	Female
D1156	49	-	-	-	Endometritis	Hospital 2	China	Female
D1157	27	-	-	-	Endometritis	Hospital 2	China	Female
D1158	47	-	-	-	Endometritis	Hospital 2	China	Female
D1159	29	-	-	-	Endometritis	Hospital 2	China	Female
D1160	43	-	-	-	Endometritis	Hospital 2	China	Female
D1161	30	-	-	-	Endometritis	Hospital 2	China	Female
D1162	38	-	-	-	Endometritis	Hospital 2	China	Female
D1163	33	-	-	-	Endometritis	Hospital 2	China	Female
D1164	45	-	-	-	Endometritis	Hospital 2	China	Female
D1165	37	-	-	-	Endometritis	Hospital 2	China	Female
D1166	40	-	-	-	Endometritis	Hospital 2	China	Female
D1167	33	-	-	-	Endometritis	Hospital 2	China	Female
D1168	41	-	-	-	Endometritis	Hospital 2	China	Female
D1169	52	-	-	-	Endometritis	Hospital 2	China	Female
D1170	62	-	-	-	Endometritis	Hospital 2	China	Female
D1171	27	-	-	-	Endometritis	Hospital 2	China	Female

D1172	46	-	-	-	Endometritis	Hospital 2	China	Female
D1173	42	-	-	-	Endometritis	Hospital 2	China	Female
D1174	39	-	-	-	Endometritis	Hospital 2	China	Female
D1175	43	-	-	-	Endometritis	Hospital 2	China	Female
D1176	54	-	-	-	Endometritis	Hospital 2	China	Female
D1177	30	-	-	-	Endometritis	Hospital 2	China	Female
D1178	35	-	-	-	Endometritis	Hospital 2	China	Female
D1179	36	-	-	-	Endometritis	Hospital 2	China	Female
D1180	37	-	-	-	Endometritis	Hospital 2	China	Female
D1181	46	-	-	-	Endometritis	Hospital 2	China	Female
D1182	48	-	-	-	Endometritis	Hospital 2	China	Female
D1183	57	-	-	-	Endometritis	Hospital 2	China	Female
D1184	25	-	-	-	Endometritis	Hospital 2	China	Female
D1185	56	-	-	-	Endometritis	Hospital 2	China	Female
D1186	46	-	-	-	Endometritis	Hospital 2	China	Female
D1187	39	-	-	-	Endometritis	Hospital 2	China	Female
D1188	40	-	-	-	Endometritis	Hospital 2	China	Female
D1189	40	-	-	-	Endometritis	Hospital 2	China	Female
D1190	38	-	-	-	Endometritis	Hospital 2	China	Female
D1191	50	-	-	-	Endometritis	Hospital 2	China	Female
D1192	40	-	-	-	Endometritis	Hospital 2	China	Female
D1193	51	-	-	-	Endometritis	Hospital 2	China	Female
D1194	45	-	-	-	Endometritis	Hospital 2	China	Female
D1195	56	-	-	-	Endometritis	Hospital 2	China	Female
D1196	63	-	-	-	Endometritis	Hospital 2	China	Female

D1197	33	-	-	-	Endometritis	Hospital 2	China	Female
D1198	60	-	-	-	Endometritis	Hospital 2	China	Female
D1199	73	-	-	-	Endometritis	Hospital 2	China	Female
D1200	32	-	-	-	Endometritis	Hospital 2	China	Female
D1201	41	-	-	-	Endometritis	Hospital 2	China	Female
D1202	40	-	-	-	Endometritis	Hospital 2	China	Female
D1203	25	-	-	-	Endometritis	Hospital 2	China	Female
D1204	58	-	-	-	Endometritis	Hospital 2	China	Female
D1205	52	-	-	-	Endometritis	Hospital 2	China	Female
D1206	32	-	-	-	Endometritis	Hospital 2	China	Female
D1207	31	-	-	-	Endometritis	Hospital 2	China	Female
D1208	43	-	-	-	Endometritis	Hospital 2	China	Female
D1209	51	-	-	-	Endometritis	Hospital 2	China	Female
D1210	46	-	-	-	Endometritis	Hospital 2	China	Female
D1211	44	-	-	-	Endometritis	Hospital 2	China	Female
D1212	66	-	-	-	Endometritis	Hospital 2	China	Female
D1213	34	-	-	-	Endometritis	Hospital 2	China	Female
D1214	68	-	-	-	Endometritis	Hospital 2	China	Female
D1215	53	-	-	-	Endometritis	Hospital 2	China	Female
D1216	32	-	-	-	Endometritis	Hospital 2	China	Female
D1217	27	-	-	-	Endometritis	Hospital 2	China	Female
D1218	62	-	-	-	Endometritis	Hospital 2	China	Female
D1219	45	-	-	-	Endometritis	Hospital 2	China	Female
D1220	44	-	-	-	Endometritis	Hospital 2	China	Female
D1221	34	-	-	-	Endometritis	Hospital 2	China	Female

D1222	35	-	-	-	Endometritis	Hospital 2	China	Female
D1223	30	-	-	-	Endometritis	Hospital 2	China	Female
D1224	46	-	-	-	Endometritis	Hospital 2	China	Female
D1225	45	-	-	-	Endometritis	Hospital 2	China	Female
D1226	32	-	-	-	Endometritis	Hospital 2	China	Female
D1227	38	-	-	-	Endometritis	Hospital 2	China	Female
D1228	29	-	-	-	Endometritis	Hospital 2	China	Female
D1229	49	-	-	-	Endometritis	Hospital 2	China	Female
D1230	49	-	-	-	Endometritis	Hospital 2	China	Female
D1231	49	-	-	-	Endometritis	Hospital 2	China	Female
D1232	28	-	-	-	Endometritis	Hospital 2	China	Female
D1233	23	-	-	-	Endometritis	Hospital 2	China	Female
D1234	46	-	-	-	Endometritis	Hospital 2	China	Female
D1235	28	-	-	-	Endometritis	Hospital 2	China	Female
D1236	47	-	-	-	Endometritis	Hospital 2	China	Female
D1237	39	-	-	-	Endometritis	Hospital 2	China	Female
D1238	54	-	-	-	Endometritis	Hospital 2	China	Female
D1239	71	-	-	-	Endometritis	Hospital 2	China	Female
D1240	51	-	-	-	Endometritis	Hospital 2	China	Female
D1241	55	-	-	-	Endometritis	Hospital 2	China	Female
D1242	33	-	-	-	Endometritis	Hospital 2	China	Female
D1243	51	-	-	-	Endometritis	Hospital 2	China	Female
D1244	35	-	-	-	Endometritis	Hospital 2	China	Female
D1245	44	-	-	-	Endometritis	Hospital 2	China	Female
D1246	36	-	-	-	Endometritis	Hospital 2	China	Female

D1247	36	-	-	-	Endometritis	Hospital 2	China	Female
D1248	48	-	-	-	Endometritis	Hospital 2	China	Female
D1249	42	-	-	-	Endometritis	Hospital 2	China	Female
D1250	22	-	-	-	Endometritis	Hospital 2	China	Female
D1251	36	-	-	-	Endometritis	Hospital 2	China	Female
D1252	36	-	-	-	Endometritis	Hospital 2	China	Female
D1253	32	-	-	-	Endometritis	Hospital 2	China	Female
D1254	50	-	-	-	Endometritis	Hospital 2	China	Female
D1255	46	-	-	-	Endometritis	Hospital 2	China	Female
D1256	32	-	-	-	Endometritis	Hospital 2	China	Female
D1257	28	-	-	-	Endometritis	Hospital 2	China	Female
D1258	41	-	-	-	Endometritis	Hospital 2	China	Female
D1259	28	-	-	-	Endometritis	Hospital 2	China	Female
D1260	29	-	-	-	Endometritis	Hospital 2	China	Female
D1261	50	-	-	-	Endometritis	Hospital 2	China	Female
D1262	52	-	-	-	Endometritis	Hospital 2	China	Female
D1263	40	-	-	-	Endometritis	Hospital 2	China	Female
D1264	55	-	-	-	Endometritis	Hospital 2	China	Female
D1265	62	-	-	-	Endometritis	Hospital 2	China	Female
D1266	49	-	-	-	Endometritis	Hospital 2	China	Female
D1267	42	-	-	-	Endometritis	Hospital 2	China	Female
D1268	40	-	-	-	Endometritis	Hospital 2	China	Female
D1269	27	-	-	-	Endometritis	Hospital 2	China	Female
D1270	36	-	-	-	Endometritis	Hospital 2	China	Female
D1271	52	-	-	-	Endometritis	Hospital 2	China	Female

D1272	57	-	-	-	Endometritis	Hospital 2	China	Female
D1273	51	-	-	-	Endometritis	Hospital 2	China	Female
D1274	62	-	-	-	Endometritis	Hospital 2	China	Female
D1275	58	-	-	-	Endometritis	Hospital 2	China	Female
D1276	39	-	-	-	Endometritis	Hospital 2	China	Female
D1277	59	-	-	-	Endometritis	Hospital 2	China	Female
D1278	69	-	-	-	Endometritis	Hospital 2	China	Female
D1279	33	-	-	-	Endometritis	Hospital 2	China	Female
D1280	60	-	-	-	Endometritis	Hospital 2	China	Female
D1281	36	-	-	-	Endometritis	Hospital 2	China	Female
D1282	39	-	-	-	Endometritis	Hospital 2	China	Female
D1283	38	-	-	-	Endometritis	Hospital 2	China	Female
D1284	53	-	-	-	Endometritis	Hospital 2	China	Female
D1285	53	-	-	-	Endometritis	Hospital 2	China	Female
D1286	55	-	-	-	Endometritis	Hospital 2	China	Female
D1287	38	-	-	-	Endometritis	Hospital 2	China	Female
D1288	47	-	-	-	Endometritis	Hospital 2	China	Female
D1289	34	-	-	-	Endometritis	Hospital 2	China	Female
D1290	65	-	-	-	Endometritis	Hospital 2	China	Female
D1291	36	-	-	-	Endometritis	Hospital 2	China	Female
D1292	25	-	-	-	Endometritis	Hospital 2	China	Female
D1293	52	-	-	-	Endometritis	Hospital 2	China	Female
D1294	31	-	-	-	Endometritis	Hospital 2	China	Female
D1295	47	-	-	-	Endometritis	Hospital 2	China	Female
D1296	48	-	-	-	Endometritis	Hospital 2	China	Female

D1297	28	-	-	-	Endometritis	Hospital 2	China	Female
D1298	47	-	-	-	Endometritis	Hospital 2	China	Female
D1299	41	-	-	-	Endometritis	Hospital 2	China	Female
D1300	33	-	-	-	Endometritis	Hospital 2	China	Female
D1301	30	-	-	-	Endometritis	Hospital 2	China	Female
D1302	35	-	-	-	Endometritis	Hospital 2	China	Female
D1303	33	-	-	-	Endometritis	Hospital 2	China	Female
D1304	47	-	-	-	Endometritis	Hospital 2	China	Female
D1305	51	-	-	-	Endometritis	Hospital 2	China	Female
D1306	43	-	-	-	Endometritis	Hospital 2	China	Female
D1307	45	-	-	-	Endometritis	Hospital 2	China	Female
D1308	48	-	-	-	Endometritis	Hospital 2	China	Female
D1309	45	-	-	-	Endometritis	Hospital 2	China	Female
D1310	38	-	-	-	Endometritis	Hospital 2	China	Female
D1311	58	-	-	-	Endometritis	Hospital 2	China	Female
D1312	41	-	-	-	Endometritis	Hospital 2	China	Female
D1313	41	-	-	-	Endometritis	Hospital 2	China	Female
D1314	31	-	-	-	Endometritis	Hospital 2	China	Female
D1315	34	-	-	-	Endometritis	Hospital 2	China	Female
D1316	34	-	-	-	Endometritis	Hospital 2	China	Female
D1317	34	-	-	-	Endometritis	Hospital 2	China	Female
D1318	41	-	-	-	Endometritis	Hospital 2	China	Female
D1319	51	-	-	-	Endometritis	Hospital 2	China	Female
D1320	39	-	-	-	Endometritis	Hospital 2	China	Female
D1321	28	-	-	-	Endometritis	Hospital 2	China	Female

D1322	31	-	-	-	Endometritis	Hospital 2	China	Female
D1323	48	-	-	-	Endometritis	Hospital 2	China	Female
D1324	48	-	-	-	Endometritis	Hospital 2	China	Female
D1325	34	-	-	-	Endometritis	Hospital 2	China	Female
D1326	36	-	-	-	Endometritis	Hospital 2	China	Female
D1327	28	-	-	-	Endometritis	Hospital 2	China	Female
D1328	55	-	-	-	Endometritis	Hospital 2	China	Female
D1329	44	-	-	-	Endometritis	Hospital 2	China	Female
D1330	34	-	-	-	Endometritis	Hospital 2	China	Female
D1331	35	-	-	-	Endometritis	Hospital 2	China	Female
D1332	43	-	-	-	Endometritis	Hospital 2	China	Female
D1333	53	-	-	-	Endometritis	Hospital 2	China	Female
D1334	59	-	-	-	Endometritis	Hospital 2	China	Female
D1335	31	-	-	-	Endometritis	Hospital 2	China	Female
D1336	48	-	-	-	Endometritis	Hospital 2	China	Female
D1337	47	-	-	-	Endometritis	Hospital 2	China	Female
D1338	49	-	-	-	Endometritis	Hospital 2	China	Female
D1339	38	-	-	-	Endometritis	Hospital 2	China	Female
D1340	36	-	-	-	Endometritis	Hospital 2	China	Female
D1341	48	-	-	-	Endometritis	Hospital 2	China	Female
D1342	37	-	-	-	Endometritis	Hospital 2	China	Female
D1343	51	-	-	-	Endometritis	Hospital 2	China	Female
D1344	30	-	-	-	Endometritis	Hospital 2	China	Female
D1345	45	-	-	-	Endometritis	Hospital 2	China	Female
D1346	40	-	-	-	Endometritis	Hospital 2	China	Female

D1347	49	-	-	-	Endometritis	Hospital 2	China	Female
D1348	44	-	-	-	Endometritis	Hospital 2	China	Female
D1349	45	-	-	-	Endometritis	Hospital 2	China	Female
D1350	70	-	-	-	Endometritis	Hospital 2	China	Female
D1351	31	-	-	-	Endometritis	Hospital 2	China	Female
D1352	52	-	-	-	Endometritis	Hospital 2	China	Female
D1353	40	-	-	-	Endometritis	Hospital 2	China	Female
D1354	51	-	-	-	Endometritis	Hospital 2	China	Female
D1355	41	-	-	-	Endometritis	Hospital 2	China	Female
D1356	57	-	-	-	Endometritis	Hospital 2	China	Female
D1357	57	-	-	-	Endometritis	Hospital 2	China	Female
H1358	26	-	-	-	Health	Hospital 2	China	Female
H1359	19	-	-	-	Health	Hospital 2	China	Female
H1360	40	-	-	-	Health	Hospital 2	China	Female
H1361	38	-	-	-	Health	Hospital 2	China	Female
H1362	39	-	-	-	Health	Hospital 2	China	Female
H1363	37	-	-	-	Health	Hospital 2	China	Female
H1364	28	-	-	-	Health	Hospital 2	China	Female
H1365	26	-	-	-	Health	Hospital 2	China	Female
H1366	27	-	-	-	Health	Hospital 2	China	Female
H1367	29	-	-	-	Health	Hospital 2	China	Female
H1368	23	-	-	-	Health	Hospital 2	China	Female
H1369	21	-	-	-	Health	Hospital 2	China	Female
H1370	38	-	-	-	Health	Hospital 2	China	Female
H1371	29	-	-	-	Health	Hospital 2	China	Female

H1372	39	-	-	-	Health	Hospital 2	China	Female
H1373	34	-	-	-	Health	Hospital 2	China	Female
H1374	44	-	-	-	Health	Hospital 2	China	Female
H1375	37	-	-	-	Health	Hospital 2	China	Female
H1376	30	-	-	-	Health	Hospital 2	China	Female
H1377	32	-	-	-	Health	Hospital 2	China	Female
H1378	29	-	-	-	Health	Hospital 2	China	Female
H1379	31	-	-	-	Health	Hospital 2	China	Female
H1380	32	-	-	-	Health	Hospital 2	China	Female
H1381	37	-	-	-	Health	Hospital 2	China	Female
H1382	35	-	-	-	Health	Hospital 2	China	Female
H1383	42	-	-	-	Health	Hospital 2	China	Female
H1384	36	-	-	-	Health	Hospital 2	China	Female
H1385	19	-	-	-	Health	Hospital 2	China	Female
H1386	34	-	-	-	Health	Hospital 2	China	Female
H1387	22	-	-	-	Health	Hospital 2	China	Female
H1388	35	-	-	-	Health	Hospital 2	China	Female
H1389	36	-	-	-	Health	Hospital 2	China	Female
H1390	39	-	-	-	Health	Hospital 2	China	Female
H1391	34	-	-	-	Health	Hospital 2	China	Female
H1392	46	-	-	-	Health	Hospital 2	China	Female
H1393	20	-	-	-	Health	Hospital 2	China	Female
H1394	33	-	-	-	Health	Hospital 2	China	Female
H1395	30	-	-	-	Health	Hospital 2	China	Female
H1396	26	-	-	-	Health	Hospital 2	China	Female

H1397	34	-	-	-	Health	Hospital 2	China	Female
H1398	33	-	-	-	Health	Hospital 2	China	Female
H1399	28	-	-	-	Health	Hospital 2	China	Female
H1400	28	-	-	-	Health	Hospital 2	China	Female
H1401	31	-	-	-	Health	Hospital 2	China	Female
H1402	26	-	-	-	Health	Hospital 2	China	Female
H1403	30	-	-	-	Health	Hospital 2	China	Female
H1404	32	-	-	-	Health	Hospital 2	China	Female
H1405	38	-	-	-	Health	Hospital 2	China	Female
H1406	37	-	-	-	Health	Hospital 2	China	Female
H1407	36	-	-	-	Health	Hospital 2	China	Female
H1408	28	-	-	-	Health	Hospital 2	China	Female
H1409	29	-	-	-	Health	Hospital 2	China	Female
H1410	38	-	-	-	Health	Hospital 2	China	Female
H1411	26	-	-	-	Health	Hospital 2	China	Female
H1412	34	-	-	-	Health	Hospital 2	China	Female
H1413	34	-	-	-	Health	Hospital 2	China	Female
H1414	35	-	-	-	Health	Hospital 2	China	Female
H1415	32	-	-	-	Health	Hospital 2	China	Female
H1416	43	-	-	-	Health	Hospital 2	China	Female
H1417	36	-	-	-	Health	Hospital 2	China	Female
H1418	41	-	-	-	Health	Hospital 2	China	Female
H1419	36	-	-	-	Health	Hospital 2	China	Female
H1420	35	-	-	-	Health	Hospital 2	China	Female
H1421	39	-	-	-	Health	Hospital 2	China	Female

H1422	37	-	-	-	Health	Hospital 2	China	Female
H1423	30	-	-	-	Health	Hospital 2	China	Female
H1424	32	-	-	-	Health	Hospital 2	China	Female
H1425	30	-	-	-	Health	Hospital 2	China	Female
H1426	29	-	-	-	Health	Hospital 2	China	Female
H1427	27	-	-	-	Health	Hospital 2	China	Female
H1428	37	-	-	-	Health	Hospital 2	China	Female
H1429	29	-	-	-	Health	Hospital 2	China	Female
H1430	35	-	-	-	Health	Hospital 2	China	Female
H1431	36	-	-	-	Health	Hospital 2	China	Female
H1432	34	-	-	-	Health	Hospital 2	China	Female
H1433	31	-	-	-	Health	Hospital 2	China	Female
H1434	28	-	-	-	Health	Hospital 2	China	Female
H1435	31	-	-	-	Health	Hospital 2	China	Female
H1436	35	-	-	-	Health	Hospital 2	China	Female
H1437	36	-	-	-	Health	Hospital 2	China	Female
H1438	34	-	-	-	Health	Hospital 2	China	Female
H1439	39	-	-	-	Health	Hospital 2	China	Female
H1440	31	-	-	-	Health	Hospital 2	China	Female
H1441	27	-	-	-	Health	Hospital 2	China	Female
H1442	29	-	-	-	Health	Hospital 2	China	Female
H1443	37	-	-	-	Health	Hospital 2	China	Female
H1444	33	-	-	-	Health	Hospital 2	China	Female
H1445	39	-	-	-	Health	Hospital 2	China	Female
H1446	27	-	-	-	Health	Hospital 2	China	Female

H1447	36	-	-	-	Health	Hospital 2	China	Female
H1448	31	-	-	-	Health	Hospital 2	China	Female
H1449	32	-	-	-	Health	Hospital 2	China	Female
H1450	37	-	-	-	Health	Hospital 2	China	Female
H1451	31	-	-	-	Health	Hospital 2	China	Female
H1452	40	-	-	-	Health	Hospital 2	China	Female
H1453	35	-	-	-	Health	Hospital 2	China	Female
H1454	30	-	-	-	Health	Hospital 2	China	Female
H1455	44	-	-	-	Health	Hospital 2	China	Female
H1456	32	-	-	-	Health	Hospital 2	China	Female
H1457	30	-	-	-	Health	Hospital 2	China	Female
H1458	22	-	-	-	Health	Hospital 2	China	Female
H1459	30	-	-	-	Health	Hospital 2	China	Female
H1460	32	-	-	-	Health	Hospital 2	China	Female
H1461	42	-	-	-	Health	Hospital 2	China	Female
H1462	36	-	-	-	Health	Hospital 2	China	Female
H1463	29	-	-	-	Health	Hospital 2	China	Female
H1464	33	-	-	-	Health	Hospital 2	China	Female
H1465	35	-	-	-	Health	Hospital 2	China	Female
H1466	28	-	-	-	Health	Hospital 2	China	Female
H1467	36	-	-	-	Health	Hospital 2	China	Female
H1468	35	-	-	-	Health	Hospital 2	China	Female
H1469	32	-	-	-	Health	Hospital 2	China	Female
H1470	31	-	-	-	Health	Hospital 2	China	Female
H1471	27	-	-	-	Health	Hospital 2	China	Female

H1472	33	-	-	-	Health	Hospital 2	China	Female
H1473	31	-	-	-	Health	Hospital 2	China	Female
H1474	30	-	-	-	Health	Hospital 2	China	Female
H1475	44	-	-	-	Health	Hospital 2	China	Female
H1476	34	-	-	-	Health	Hospital 2	China	Female
H1477	36	-	-	-	Health	Hospital 2	China	Female
H1478	30	-	-	-	Health	Hospital 2	China	Female
H1479	29	-	-	-	Health	Hospital 2	China	Female
H1480	30	-	-	-	Health	Hospital 2	China	Female
H1481	41	-	-	-	Health	Hospital 2	China	Female
H1482	20	-	-	-	Health	Hospital 2	China	Female
H1483	35	-	-	-	Health	Hospital 2	China	Female
H1484	34	-	-	-	Health	Hospital 2	China	Female
H1485	26	-	-	-	Health	Hospital 2	China	Female
H1486	40	-	-	-	Health	Hospital 2	China	Female
H1487	34	-	-	-	Health	Hospital 2	China	Female
H1488	29	-	-	-	Health	Hospital 2	China	Female
H1489	22	-	-	-	Health	Hospital 2	China	Female
H1490	32	-	-	-	Health	Hospital 2	China	Female
H1491	31	-	-	-	Health	Hospital 2	China	Female
H1492	37	-	-	-	Health	Hospital 2	China	Female
H1493	24	-	-	-	Health	Hospital 2	China	Female
H1494	37	-	-	-	Health	Hospital 2	China	Female
H1495	38	-	-	-	Health	Hospital 2	China	Female
H1496	36	-	-	-	Health	Hospital 2	China	Female

H1497	35	-	-	-	Health	Hospital 2	China	Female
H1498	34	-	-	-	Health	Hospital 2	China	Female
H1499	33	-	-	-	Health	Hospital 2	China	Female
H1500	32	-	-	-	Health	Hospital 2	China	Female
H1501	30	-	-	-	Health	Hospital 2	China	Female
H1502	38	-	-	-	Health	Hospital 2	China	Female
H1503	28	-	-	-	Health	Hospital 2	China	Female
H1504	36	-	-	-	Health	Hospital 2	China	Female
H1505	32	-	-	-	Health	Hospital 2	China	Female
H1506	30	-	-	-	Health	Hospital 2	China	Female
H1507	28	-	-	-	Health	Hospital 2	China	Female
H1508	39	-	-	-	Health	Hospital 2	China	Female
H1509	23	-	-	-	Health	Hospital 2	China	Female
H1510	33	-	-	-	Health	Hospital 2	China	Female
H1511	34	-	-	-	Health	Hospital 2	China	Female
H1512	31	-	-	-	Health	Hospital 2	China	Female
H1513	29	-	-	-	Health	Hospital 2	China	Female
H1514	33	-	-	-	Health	Hospital 2	China	Female
H1515	33	-	-	-	Health	Hospital 2	China	Female
H1516	43	-	-	-	Health	Hospital 2	China	Female
H1517	33	-	-	-	Health	Hospital 2	China	Female
H1518	36	-	-	-	Health	Hospital 2	China	Female
H1519	23	-	-	-	Health	Hospital 2	China	Female
H1520	24	-	-	-	Health	Hospital 2	China	Female
H1521	24	-	-	-	Health	Hospital 2	China	Female

H1522	23	-	-	-	Health	Hospital 2	China	Female
H1523	23	-	-	-	Health	Hospital 2	China	Female
H1524	27	-	-	-	Health	Hospital 2	China	Female
H1525	30	-	-	-	Health	Hospital 2	China	Female
H1526	28	-	-	-	Health	Hospital 2	China	Female
H1527	24	-	-	-	Health	Hospital 2	China	Female
H1528	25	-	-	-	Health	Hospital 2	China	Female
H1529	44	-	-	-	Health	Hospital 2	China	Female
H1530	28	-	-	-	Health	Hospital 2	China	Female
H1531	25	-	-	-	Health	Hospital 2	China	Female
H1532	31	-	-	-	Health	Hospital 2	China	Female
H1533	25	-	-	-	Health	Hospital 2	China	Female
H1534	39	-	-	-	Health	Hospital 2	China	Female
H1535	22	-	-	-	Health	Hospital 2	China	Female
H1536	33	-	-	-	Health	Hospital 2	China	Female
H1537	32	-	-	-	Health	Hospital 2	China	Female
H1538	39	-	-	-	Health	Hospital 2	China	Female
H1539	35	-	-	-	Health	Hospital 2	China	Female
H1540	25	-	-	-	Health	Hospital 2	China	Female
H1541	40	-	-	-	Health	Hospital 2	China	Female
H1542	27	-	-	-	Health	Hospital 2	China	Female
H1543	29	-	-	-	Health	Hospital 2	China	Female
H1544	29	-	-	-	Health	Hospital 2	China	Female
H1545	26	-	-	-	Health	Hospital 2	China	Female
H1546	28	-	-	-	Health	Hospital 2	China	Female

H1547	25	-	-	-	Health	Hospital 2	China	Female
H1548	31	-	-	-	Health	Hospital 2	China	Female
H1549	29	-	-	-	Health	Hospital 2	China	Female
H1550	32	-	-	-	Health	Hospital 2	China	Female
H1551	39	-	-	-	Health	Hospital 2	China	Female
H1552	39	-	-	-	Health	Hospital 2	China	Female
H1553	31	-	-	-	Health	Hospital 2	China	Female
H1554	35	-	-	-	Health	Hospital 2	China	Female
H1555	32	-	-	-	Health	Hospital 2	China	Female
H1556	35	-	-	-	Health	Hospital 2	China	Female
H1557	31	-	-	-	Health	Hospital 2	China	Female
H1558	30	-	-	-	Health	Hospital 2	China	Female
H1559	37	-	-	-	Health	Hospital 2	China	Female
H1560	32	-	-	-	Health	Hospital 2	China	Female
H1561	35	-	-	-	Health	Hospital 2	China	Female
H1562	33	-	-	-	Health	Hospital 2	China	Female
H1563	34	-	-	-	Health	Hospital 2	China	Female
H1564	40	-	-	-	Health	Hospital 2	China	Female
H1565	35	-	-	-	Health	Hospital 2	China	Female
H1566	36	-	-	-	Health	Hospital 2	China	Female
H1567	31	-	-	-	Health	Hospital 2	China	Female
H1568	28	-	-	-	Health	Hospital 2	China	Female
H1569	33	-	-	-	Health	Hospital 2	China	Female
H1570	32	-	-	-	Health	Hospital 2	China	Female
H1571	33	-	-	-	Health	Hospital 2	China	Female

H1572	30	-	-	-	Health	Hospital 2	China	Female
H1573	39	-	-	-	Health	Hospital 2	China	Female
H1574	40	-	-	-	Health	Hospital 2	China	Female
H1575	29	-	-	-	Health	Hospital 2	China	Female
H1576	33	-	-	-	Health	Hospital 2	China	Female
H1577	31	-	-	-	Health	Hospital 2	China	Female
H1578	36	-	-	-	Health	Hospital 2	China	Female
H1579	29	-	-	-	Health	Hospital 2	China	Female
H1580	40	-	-	-	Health	Hospital 2	China	Female
H1581	36	-	-	-	Health	Hospital 2	China	Female
H1582	44	-	-	-	Health	Hospital 2	China	Female
H1583	36	-	-	-	Health	Hospital 2	China	Female
H1584	40	-	-	-	Health	Hospital 2	China	Female
H1585	29	-	-	-	Health	Hospital 2	China	Female
H1586	36	-	-	-	Health	Hospital 2	China	Female
H1587	34	-	-	-	Health	Hospital 2	China	Female
H1588	41	-	-	-	Health	Hospital 2	China	Female
H1589	30	-	-	-	Health	Hospital 2	China	Female
H1590	37	-	-	-	Health	Hospital 2	China	Female
H1591	31	-	-	-	Health	Hospital 2	China	Female
H1592	32	-	-	-	Health	Hospital 2	China	Female
H1593	40	-	-	-	Health	Hospital 2	China	Female
H1594	39	-	-	-	Health	Hospital 2	China	Female
H1595	35	-	-	-	Health	Hospital 2	China	Female
H1596	47	-	-	-	Health	Hospital 2	China	Female

H1597	30	-	-	-	Health	Hospital 2	China	Female
H1598	36	-	-	-	Health	Hospital 2	China	Female
H1599	34	-	-	-	Health	Hospital 2	China	Female
H1600	36	-	-	-	Health	Hospital 2	China	Female
H1601	36	-	-	-	Health	Hospital 2	China	Female
H1602	36	-	-	-	Health	Hospital 2	China	Female
H1603	40	-	-	-	Health	Hospital 2	China	Female
H1604	40	-	-	-	Health	Hospital 2	China	Female
H1605	36	-	-	-	Health	Hospital 2	China	Female
H1606	36	-	-	-	Health	Hospital 2	China	Female
H1607	31	-	-	-	Health	Hospital 2	China	Female
H1608	39	-	-	-	Health	Hospital 2	China	Female
H1609	33	-	-	-	Health	Hospital 2	China	Female
H1610	45	-	-	-	Health	Hospital 2	China	Female
H1611	35	-	-	-	Health	Hospital 2	China	Female
H1612	31	-	-	-	Health	Hospital 2	China	Female

Supplementary Table 3 Summary of the public sequencing data used in this study.

Project ID	Data type	# women	# uterine samples	# vaginal samples	Country	Ethnicity	Age	Menstrual phase	Diagnosis
SRP064295	16S V3-V5	5	19	7	USA	Caucasian	46-59	Not provided	Benign disease
PRJEB14941	16S V4	56	0	288	Tanzania	Not provided	18-39	Not provided	Pregnancy
PRJEB16013	16S V4-V5	91	81	90	China	Not provided	22-48	Proliferative 70; Secretary 12	Hysteromyoma 18; Endometrial polyps 11; Endometriosis 68
PRJEB24147	16S V4-V5	36	0	37	China	Not provided	22-45	Proliferative 25; Secretary 5	Hysteromyoma 8; Endometrial polyps 6; Endometriosis 22
PRJNA481576	16S V3-V5	75	208	152	USA	Caucasian 58; Mongolian 2; Not provided 15	29-83	Not provided	Benign disease
PRJNA547595	16S V4	16	0	79	USA	Caucasian 15; Other 1	15-57	Not provided	Not provided

Supplementary Table 4 The primers used in qPCR analysis.

Target	Primer	Sequence (5'-3')	Target	Primer	Sequence (5'-3')
TNF-α	Forward	CTGTGCCTCAGCCTCTTCTC	*Clostridium*	Forward	TTGCTGTGTTAGAAGTGAATGAAGG
	Reverse	ACTGATGAGAGGGAGCCCAT		Reverse	TCCTAACTTCATGATTTTCCATTCC
IL1β	Forward	ACTGATGAGAGGGAGCCCAT	*Prevotella*	Forward	ACTAGCAATTAGAGTCGAGCGA
	Reverse	CTCCACGGGCAAGACATAGG		Reverse	TGCATCTACCCGAAGCTTATCG
IL-17	Forward	GAAGTTGGACCACCACATGA	*Lactobacillus*	Forward	GCAATGATGCGTAGCCGAAC
	Reverse	TCCCTCTTCAGGACCAGGAT		Reverse	GCACTTCTTCTCTAACAACAGGG
CXCL5	Forward	TGGCATTTCTGCTGCTGTT	16S RNA	1369F	CGGTGAATACGTTCYCGG
	Reverse	TGCATTCCGCTTTGTTTTC		1492R	GGWTACCTTGTTACGACTT
CD38	Forward	GACGCTGCCTGATCTATACTCAA	*Clostridium* for genus level	Forward	CGGTGAAATGCGTAGAKATTA
	Reverse	GTGCGTAGTCTTCATTGGTGATG		Reverse	CGAATTAAACCACATGCTCCG
CD138	Forward	GTGTGTTGTCTCTGAGTTTGTCG	*Prevotella* for genus level	Forward	CCAGCCAAGTAGCGTGCA
	Reverse	CAGGCTCTTCCAATGTCACAAAG		Reverse	TGGACCTTCCGTATTACCGC
GAPDH	Forward	AACAGCAACTCCCACTCTTC	*Lactobacillus* for genus level	Forward	CTCAAAACTAAACAAAGTTTC
	Reverse	CCTCTCTTGCTCAGTGTCCT		Reverse	CTTGTACACACCGCCCGTCA

Supplementary Table 5 Probes used for fluorescence in situ hybridization.

Probe	Sequence (5’-3’)
EUB338I	GCTGCCTCCCGTAGGAGT
EUB338II	GCAGCCACCCGTAGGTGT
EUB338III	GCTGCCACCCGTAGGTGT
NON338	ACTCCTACGGGAGGCAGC
Clostridium-specific	CCTTTGGTTGAATGATGATG
Prevotella-specific	GCTATCTATTCGCTGCCCCTC
