# Supplementary material for: Translocation of vaginal microbiota is involved in impairment and protection of uterine health
Source: Nat Commun. 2021 Jul 7;12:4191. doi: 10.1038/s41467-021-24516-8 (PMC8263591; doi:10.1038/s41467-021-24516-8)
Supplement: Supplementary file 3 — Reporting Summary [file 41467_2021_24516_MOESM3_ESM.pdf]

nature research

Corresponding author(s): Fangqing Zhao
Last updated by author(s): Jun 10, 2021

Reporting Summary

Nature Research wishes to improve the reproducibility of the work that we publish. This form provides structure for consistency and transparency in reporting. For further information on Nature Research policies, see our Editorial Policies and the Editorial Policy Checklist.

Statistics

For all statistical analyses, confirm that the following items are present in the figure legend, table legend, main text, or Methods section.

n/a	Confirmed	
☐	☒	The exact sample size (n) for each experimental group/condition, given as a discrete number and unit of measurement
☐	☒	A statement on whether measurements were taken from distinct samples or whether the same sample was measured repeatedly
☐	☒	The statistical test(s) used AND whether they are one- or two-sided<br>*Only common tests should be described solely by name; describe more complex techniques in the Methods section.*
☐	☒	A description of all covariates tested
☐	☒	A description of any assumptions or corrections, such as tests of normality and adjustment for multiple comparisons
☐	☒	A full description of the statistical parameters including central tendency (e.g. means) or other basic estimates (e.g. regression coefficient) AND variation (e.g. standard deviation) or associated estimates of uncertainty (e.g. confidence intervals)
☐	☒	For null hypothesis testing, the test statistic (e.g. F, t, r) with confidence intervals, effect sizes, degrees of freedom and P value noted<br>*Give P values as exact values whenever suitable.*
☒	☐	For Bayesian analysis, information on the choice of priors and Markov chain Monte Carlo settings
☒	☐	For hierarchical and complex designs, identification of the appropriate level for tests and full reporting of outcomes
☐	☒	Estimates of effect sizes (e.g. Cohen's d, Pearson's r), indicating how they were calculated

Our web collection on statistics for biologists contains articles on many of the points above.

Software and code

Policy information about availability of computer code

Data collection	No software was used.

Data analysis	The V4 regions of the 16S rRNA gene amplicon were marked by mapping the 515F and 806R primers against the reference sequences using Bowtie 2 v2.4.1. Subsequently, we merged the PE reads to form long sequences and mapped them to the reference sequences to capture the sequences falling into the marked V4 regions. A quality filtering step was applied according to the Phred scores via a script split_libraries_fastq.py (–r 3 –p 0.75 –q 3 –n 0) in Quantitative Insights into Microbial Ecology (QIIME) v1.9.1. Chimeric sequences were identified and removed using two QIIME commands, identify_chimeric_seqs.py and filter_fasta.py (usearch61 option that runs the UCHIME algorithm). High-quality reads were merged into long sequences by the overlaps of each pair of PE-reads using FLASH v1.2.11 with the implementation of default options. The merged long sequences containing 'N' were discarded, and operational taxonomic units (OTUs) were identified from the trimmed sequences with a 97% sequence identity threshold using UCLUST in QIIME. A representative sequence was picked for each OTU and the Greengenes database was used to generate an OTU table with taxonomy. Bray-Curtis (BC) distance and unweighted UniFrac distance computation, and inter-group comparisons were conducted using Wilcoxon rank-sum tests and PERMANOVA in R package vegan v2.5-6. The phylogenetic tree used to calculate UniFrac distance was created using FastTree v2.1.10. Pairwise correlation coefficients between the uterine and vaginal bacteria were computed at the OTU and the genus level respectively using SparCC with 100 bootstraps to estimate P values. Source tracking of uterine and vaginal microbiome within the same subject was performed based on 16S rRNA taxonomic data by using SourceTracker v1.0. Raw reads of metagenomic sequencing were filtered using the FASTQ quality filter in the FASTX-toolkit v0.0.14 with the parameters of –p 90 –q 25 –Q33. High-quality reads were first aligned to the hg38 release of the human reference genome using the BWA v0.7.17 algorithm with default parameters. The metagenomic read pairs were used for strain-level source tracking as follows: Uterus-vagina paired samples from the same subject were aligned to MetaPhlAn v2.7.6 species-specific gene markers to measure microbes shared by two body sites at the strain level. The "mpileup" feature in SAMtools v0.1.19 was used to calculate coverage and determine variable sites for each marker gene. The statistical analysis of the animal experiments was performed using the Graphpad Prism v8.0 software. The code scripts used for data processing, analysis, and visualization have been deposited to Zenodo under https://doi.org/10.5281/zenodo.4925167.

For manuscripts utilizing custom algorithms or software that are central to the research but not yet described in published literature, software must be made available to editors and reviewers. We strongly encourage code deposition in a community repository (e.g. GitHub). See the Nature Research guidelines for submitting code & software for further information.

Data

Policy information about availability of data

All manuscripts must include a data availability statement. This statement should provide the following information, where applicable:

- Accession codes, unique identifiers, or web links for publicly available datasets
- A list of figures that have associated raw data
- A description of any restrictions on data availability

The sequencing data generated in this study have been deposited in the NCBI SRA database under accession number (PRJNA737052). The public 16S rRNA sequences used in this study are available in the NCBI SRA database under accession number SRP064295 (https://www.ncbi.nlm.nih.gov/sra/?term=SRP064295), PRJEB14941 (https://www.ncbi.nlm.nih.gov/sra/?term=PRJEB14941), PRJEB16013 (https://www.ncbi.nlm.nih.gov/sra/?term=PRJEB16013), PRJEB24147 (https://www.ncbi.nlm.nih.gov/sra/?term=PRJEB24147), PRJNA481576 (https://www.ncbi.nlm.nih.gov/sra/?term=PRJNA481576), and PRJNA547595 (https://www.ncbi.nlm.nih.gov/sra/?term=PRJNA547595). The reference sequences of the 16S rRNA genes are available in the SILVA rRNA database (https://www.arb-silva.de) and the Greengenes database (http://greengenes.secondgenome.com). The reference genomes of Clostridium and Prevotella strains are available in the Human Microbiome Project (https://www.hmpdacc.org). Source data are provided as a Source Data file.

Field-specific reporting

Please select the one below that is the best fit for your research. If you are not sure, read the appropriate sections before making your selection.

☒ Life sciences ☐ Behavioural & social sciences ☐ Ecological, evolutionary & environmental sciences

For a reference copy of the document with all sections, see nature.com/documents/nr-reporting-summary-flat.pdf

Life sciences study design

All studies must disclose on these points even when the disclosure is negative.

Sample size	No sample size calculation was performed. The sample sizes were chosen depending on how many samples in each comparison group could be collected, successfully sequenced, and passed quality control. Using >5 animals are well accepted in animal experiments.
Data exclusions	Women with abnormal leucorrhea, cervicitis, abnormal levels of sex hormones, TCT- or HPV- positive, malignant tumors, autoimmune disease, severe metabolic diseases, mental disorders, and those who had been administered vaginal medication, antibiotic treatment, or hormonal drugs within 3 months, or engaged in sexual activity or vaginal flushing 7 days before hospital visits were excluded from the study to avoid interfering with the results of data analysis.
Replication	There is an interval of one month between two replicates, all attempts were successful.
Randomization	Female participants were randomly recruited during their visits at the gynecological clinics of the Aviation General Hospital. All participants were suspected of having intrauterine lesions but later some of them were ruled out based on hysteroscopy examination by a skilled endoscopist. The diagnosis of chronic endometritis was made according to the following criteria: the presence of stromal edema, focal or diffuse periglandular hyperemia, and micropolyps <1 mm in size. Accordingly, the participants were allocated into two groups of with and without endometritis.
Blinding	The doctors collecting uterine and vaginal samples, laboratory staffs operating sequencing and investigators processing raw sequencing data were blinded to group allocation.

Reporting for specific materials, systems and methods

We require information from authors about some types of materials, experimental systems and methods used in many studies. Here, indicate whether each material, system or method listed is relevant to your study. If you are not sure if a list item applies to your research, read the appropriate section before selecting a response.

Materials & experimental systems

n/a	Involved in the study
☐	☒ Antibodies
☒	☐ Eukaryotic cell lines
☒	☐ Palaeontology and archaeology
☐	☒ Animals and other organisms
☐	☒ Human research participants
☒	☐ Clinical data
☒	☐ Dual use research of concern

Methods

n/a	Involved in the study
☒	☐ ChIP-seq
☒	☐ Flow cytometry
☒	☐ MRI-based neuroimaging

Antibodies

Antibodies used

anti-TNF-α (1:50, Cat# ab109322, Abcam, Cambridge, MA)
anti-CD38 (1:100, bs-0979R, Bioss Antibodies, Beijing, China)
Alexa Fluor-488 donkey anti-rabbit IgG (H + L) (1:500, A21206, Invitrogen)
Alexa Fluor-594 goat Anti-rabbit IgG (H+L), F(ab')2 Fragment (1:500, 8889S, CST)

Validation

The antibodies used in our study were validated by the manufacturers and used according to the manufacturers' instructions. For certain applications, antibodies have been re-validated by titrating their concentrations and evaluating the staining efficiency in comparison with positive controls and negative controls.
anti-TNF-α
Reference: Tumor necrosis factor receptor-2 signaling pathways promote survival of cancer stem-like CD133+ cells in clear cell renal carcinoma. FASEB Bioadv. 2020;2(2):126-144.
anti-CD38
Reference: The P387 thrombospondin-4 variant promotes accumulation of macrophages in atherosclerotic lesions. FASEB J. 2020 Sep;34(9):11529-11545.
Alexa Fluor-488 donkey anti-rabbit IgG (H + L)
Reference: Biglycan Regulates MG63 Osteosarcoma Cell Growth Through a LPR6/β-Catenin/IGFR-IR Signaling Axis. Front Oncol. 2018 Oct 23;8:470.
Alexa Fluor-594 goat Anti-rabbit IgG (H+L), F(ab')2 Fragment
Reference: Calreticulin enhances the secretory trafficking of a misfolded α-1-antitrypsin. J Biol Chem. 2020 Dec 4;295 (49):16754-16772.

Animals and other organisms

Policy information about studies involving animals; ARRIVE guidelines recommended for reporting animal research

Laboratory animals

Nine months old female Brown Norway and Sprague Dawley rats.

Wild animals

The study did not involve wild animals.

Field-collected samples

The study did not involve samples collected from the field.

Ethics oversight

The study was approved by the Ethical Committee of the Aviation General Hospital of China Medical University and performed according to the principles expressed in the Declaration of Helsinki.

Note that full information on the approval of the study protocol must also be provided in the manuscript.

Human research participants

Policy information about studies involving human research participants

Population characteristics

Women with abnormal leucorrhea, cervicitis, abnormal levels of sex hormones, TCT- or HPV- positive, malignant tumors, autoimmune disease, severe metabolic diseases, mental disorders, and those who had been administered vaginal medication, antibiotic treatment, or hormonal drugs within 3 months, or engaged in sexual activity or vaginal flushing 7 days before hospital visits were excluded from the study. We recruited 145 women aged 19-71 years in the first cohort of our study, 106 of whom had at least one abortion and 95 women (72 vaginal delivery vs 23 cesarean sections) with the records of delivery mode.

Recruitment

Female participants were randomly recruited during their visits at the gynecological clinics of the Aviation General Hospital. This study only divided the participants into healthy and diseased groups based on diagnostic criteria, so there may be a

	spectrum effect and the discriminatory bacterial taxa of the uterine and vaginal microbiota of the women suffering from chronic endometritis may not be entirely accurate. In addition, many clinical and physiological information about the literacy level, daily health care status, and lifestyle habits of both the women we recruited and the volunteers in the public data were not available, and these factors may introduce self-selection bias that would affect the results of the correlation analysis between age and microbiota dynamics.
Ethics oversight	The study was approved by the Ethical Committee of the Aviation General Hospital of China Medical University and performed according to the principles expressed in the Declaration of Helsinki. Written informed consent was obtained from each participant.

Note that full information on the approval of the study protocol must also be provided in the manuscript.
